# Nanomedicine Approaches for Autophagy Modulation in Cancer Therapy

**DOI:** 10.1002/smsc.202400607

**Published:** 2025-04-11

**Authors:** Sohaib Mahri, Rodolfo Villa, Ya‐Ping Shiau, Menghuan Tang, Kelsey Jane Racacho, Qiufang Zong, Saiful Islam Chowdhury, Tan Hua, Felipe Godinez, Andrew Birkeland, Tzu‐Yin Lin, Yuanpei Li

**Affiliations:** ^1^ Department of Biochemistry and Molecular Medicine UC Davis Comprehensive Cancer Center University of California Davis Sacramento CA 95817 USA; ^2^ Department of Radiology University of California Davis Sacramento CA 95817 USA; ^3^ Department of Otolaryngology, Head and Neck Surgery University of California Davis Sacramento CA 95817 USA; ^4^ Division of Hematology/Oncology Department of Internal Medicine University of California Davis Sacramento CA 95817 USA

**Keywords:** autophagy inhibitions, autophagy modulations, cancer therapies, clinical translations, drug resistances, nanomedicines

## Abstract

Cancer is a daunting global health problem with a steadily rising incidence. Despite the wide arsenal of current anticancer therapies, challenges such as drug resistance, tumor heterogeneity, poor targeting, and severe side effects often lead to suboptimal efficacy and poor patient outcomes, highlighting the need for innovative therapies. Autophagy modulation has emerged as an attractive approach to complement existing therapies. The dual role of autophagy in cancer promotion and suppression has inspired the development of new drugs and therapeutic strategies focusing on both inhibition and induction. Despite the promising results of current autophagy modulators in preclinical studies, challenges such as the lack of selectivity and potency, toxicity, poor pharmacokinetics, and inadequate tumor targeting continue to limit their successful clinical translation. Many of these challenges could be overcome using nanomedicine. This review explores recent advancements in nanomedicine strategies for autophagy modulation. Successful combination strategies leveraging nanoparticles and autophagy modulators in synergy with chemotherapy, immunotherapy, phototherapy, gene therapy, and other modalities are presented. Additionally, nanomaterials with intrinsic autophagy‐modulating capabilities, such as self‐assembling autophagy inhibitors, are discussed. Finally, limitations of autophagy modulators currently in clinical trials are discussed, and future perspectives on designing nanomedicine for successful clinical implementation are explored.

## Graphical abstract

Autophagy modulation holds promise for enhancing cancer treatment, yet clinical translation remains challenging. This review explores nanomedicine‐based strategies to improve autophagy‐targeting drugs, highlighting their synergy with chemotherapy, immunotherapy, phototherapy, and gene therapy. It also discusses nanoparticle design and clinical progress, offering insights into future directions for optimizing cancer therapeutics.

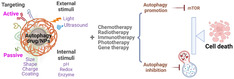

## 1 Introduction

Cancer is one of the leading causes of mortality worldwide, accounting for 20 million new cases in 2022, with a projected 77% increase by 2050.^[^ ^1^ ^]^ Despite tremendous progress in prevention, early diagnosis, and treatment, cancer remains a formidable challenge due to its complex biology, heterogeneity, and adaptability. Current anticancer therapies are often faced with drug resistance, limited drug targeting, and severe side effects, contributing to treatment failure and poor outcomes.^[^ ^2^, ^3^ ^]^ This highlights the urgent need for innovative therapeutic strategies to target cancer effectively.

One promising area of research for cancer therapy is targeting autophagy. Autophagy is a highly conserved cellular process for degrading and recycling damaged organelles and proteins.^[^ ^4^ ^]^ It is essential for cell homeostasis and survival. It plays a complex role in cancer initiation, progression, and metastasis, with evidence of its involvement in cancer cell migration, invasiveness, epithelial‐to‐mesenchymal transition, and anoikis.^[^ ^5^, ^6^ ^]^ The dual role of autophagy in tumor suppression and promotion is widely acknowledged, highlighting its importance in the complex dynamics of cancer development and therapy.^[^ ^7^, ^8^ ^]^ Broadly speaking, autophagy contributes to tumor suppression at early stages while promoting tumor growth at later stages.^[^ ^6^ ^]^ As a tumor suppressor, autophagy inhibits tumor initiation and transformation by preventing the accumulation of damaged organelles and proteins—thus preventing genomic instability and cellular stress—and enhancing immunosurveillance.^[^ ^8^ ^]^ In more established tumors, autophagy assumes a cytoprotective role by supplying nutrients to cancer cells, facilitating immune escape and metastasis, and promoting resistance to chemotherapy and other treatments.^[^ ^8^ ^]^ With varying success, this duality has been leveraged to develop therapeutic strategies to improve cancer treatments and overcome drug resistance.

Nanomedicine has emerged as a transformative approach to overcome translational hurdles and enhance the efficacy and safety of drugs for treating cancer and other diseases.^[^ ^9^, ^10^ ^]^ Through decades of innovation and validation, numerous nanosystems have been successfully leveraged to achieve tangible improvements over conventional therapies. Key advancements include improved formulation of poorly soluble drugs, superior pharmacokinetics via prolonged blood circulation time, increased drug accumulation in tumor tissues through active or passive targeting, protection of active drugs, reduced toxicity, and the ability to coadminister multiple therapeutics and achieve controlled release in vivo.^[^ ^11^, ^12^, ^13^, ^14^ ^]^ Conventional and novel nanoparticle (NP) delivery systems have been used as carriers to deliver autophagy‐modulating drugs, either as single agents or in combination with other therapies. Certain NPs (e.g., iron oxide, gold, and silica NPs) possess inherent autophagy‐modulating properties and can serve as standalone agents, although combination strategies often offer greater therapeutic success.

This review explores the intersection between autophagy modulation and nanomedicine in cancer therapy, highlighting recent advancements in the field. It covers the fundamental biology of autophagy and its implications for cancer treatment. Conventional and novel autophagy modulators and their combination with other modalities such as chemotherapy, immunotherapy, phototherapy, and gene therapy are discussed. Finally, the review examines autophagy modulators in clinical trials, identifies the limitations of current treatments, and explores emerging trends and nanomedicine‐based designs to accelerate clinical translation.

## 2 Autophagy

### 2.1 Introduction to Autophagy

Autophagy, from the Greek meaning “to eat” (phagy) and “oneself” (auto), is a conserved cellular process that degrades and recycles damaged organelles, misfolded proteins, and other cellular components. It serves both as a quality control mechanism and as a source of energy during times of stress.^[^ ^15^ ^]^ Several factors can induce autophagy, including nutrient deprivation, hypoxia, oxidative stress, endoplasmic reticulum (ER) stress, mitochondrial dysfunction, infections, hormonal changes (e.g., mTOR inhibition), and chemotherapeutic agents.^[^ ^16^ ^]^ These stimuli activate autophagy to promote cell survival, maintain homeostasis, and help clear damaged components.^[^ ^17^, ^18^, ^19^ ^]^

The concept of autophagy emerged in the 1960s following Christian de Duve's discovery of lysosomes, which are critical for cellular degradation processes. It was first observed that cells could degrade their own components by enclosing them in membranes to form vesicles, which were then transported to lysosomes for degradation.^[^ ^20^ ^]^ It was not until the early 1990s that Yoshinori Ohsumi conducted pioneering experiments on *S. cerevisiae* to identify essential genes involved in autophagy.^[^ ^20^ ^]^ His work uncovered the fundamental machinery of autophagy, such as the role of the Apg12 conjugation system.^[^ ^21^, ^22^, ^23^ ^]^ The late 1990s saw the discovery of Beclin‐1, an essential autophagy gene that is monoallelically deleted in 40–75% of ovarian, breast, and prostate cancer,^[^ ^24^ ^]^ providing the first evidence linking autophagy defects and cancer development. While initially thought of as a tumor suppressor, it has since been recognized that autophagy plays a complex role in cancer, acting as both a suppressor in early stages and a promoter in advanced stages.^[^ ^25^, ^26^ ^]^

**Figure 1:**
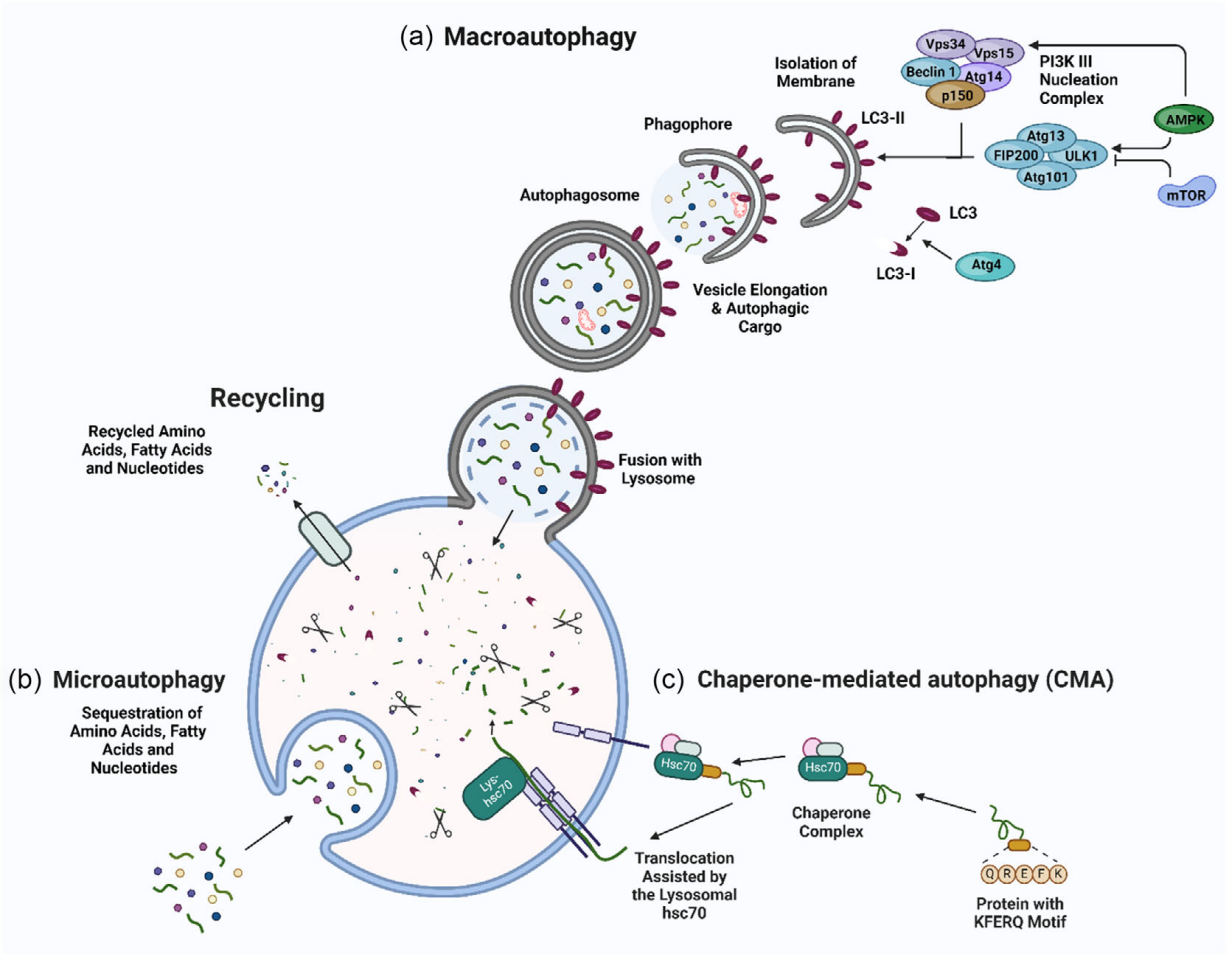
Autophagy pathways. a) In macroautophagy, cytoplasmic cargo, including damaged organelles and proteins, are enclosed in a double‐membrane vesicle (autophagosome) and delivered to lysosomes for degradation and recycling. b) In microautophagy, lysosomes take the cargo up directly through membrane protrusions and infoldings, where degradation occurs. c) CMA utilizes the Hsc70 to recognize cytosolic proteins containing KFERQ‐like motifs; the complex is then delivered into lysosomes through LAMP2A. Macroautophagy begins with forming a phagophore, which originates from membrane sources in the ER. This membrane elongates and curves around targeted cytoplasmic material, forming a double‐membrane structure known as the autophagosome. Once complete, the autophagosome fuses with a lysosome, where the inner membrane and its cargo are exposed to lysosomal hydrolases for degradation. Adapted under the terms of the CC BY license.^[^ ^454^, ^455^ ^]^ Copyright 2022, 2023, The Authors. Published by Wiley. Created in BioRender. Racacho, K. (2024) https://BioRender.com/t25y362.

There are three main types of autophagy: macroautophagy, microautophagy, and chaperon‐mediated autophagy (CMA), each with distinct morphological mechanisms to deliver cargo to the lysosome (**Figure** **1**).^[^ ^15^, ^27^ ^]^ Macroautophagy involves the formation of a double‐membraned autophagosome that engulfs cytoplasmic material before fusing with the lysosome. In contrast, microautophagy involves the direct engulfment of cytoplasmic material through lysosomal membrane protrusions or invaginations, bypassing the need for autophagosomes. While typically nonselective, it can selectively target specific structures such as peroxisomes or parts of the nucleus.^[^ ^28^ ^]^ CMA utilizes a receptor‐mediated translocation complex, LAMP‐2A, to selectively import proteins across the lysosomal membrane.^[^ ^15^, ^27^, ^29^ ^]^

Macroautophagy activity is regulated by the mechanistic target of rapamycin complex 1 (mTORC1) and AMP‐activated protein kinase (AMPK). Autophagosome formation is initiated by the assembly of the ULK1 and ULK2 (unc‐51‐like kinase 1 and 2) complex, which includes the autophagy‐related proteins ATG13, ATG101, and FIP200. ATG101 localizes to the phagophore, stabilizes the expression of ATG13, and assists the complex in assembling the ULK/ATG1 complex.^[^ ^19^, ^30^, ^31^, ^32^, ^33^ ^]^ The phagophore's nucleation and expansion depend on the class III phosphatidylinositol 3‐kinase (PI3K) complex, which includes hVps34 and Beclin‐1.^[^ ^34^, ^35^ ^]^ The Beclin‐1: hVps34 complex facilitates the recruitment of the ATG12‐ATG5 multimeric complex and the lipidated form of LC3 (microtubule‐associated protein 1A/1B‐light chain), a marker for autophagosome formation. p62 (SQSTM1) functions as an autophagy adaptor by binding ubiquitinated cargo and interacting with LC3 on autophagosomes, facilitating cargo recruitment into autophagosomes for lysosomal degradation.^[^ ^36^, ^37^ ^]^ Once the autophagosome is completed, the ATG12‐ATG5 complex dissociates, and LC3 on the cytosolic surface of the autophagosome is cleaved from phosphatidylethanolamine by ATG4.^[^ ^34^, ^38^ ^]^ The final stage of autophagy involves the release of metabolites from lysosomal degradation into the cytosol.

Microautophagy involves the endosomal sorting complex required for transport (ESCRT complex), specifically ESCRT‐III. This complex is crucial for membrane cleavage, allowing vesicles with cytosolic material to bud into the lysosome.^[^ ^39^ ^]^ ESCRT‐III complex proteins, such as Snf7, Vps4, and Vps20, assemble and drive the engulfment of the lysosomal membrane. Additionally, ATG7 can regulate autophagy through interactions with other autophagy‐related proteins.^[^ ^40^ ^]^

CMA is a selective process that targets individual proteins for degradation. Proteins destined for CMA degradation contain a KFERQ‐like peptide motif, which is recognized by the cytosolic chaperone Hsc70 (heat shock cognate protein 70).^[^ ^41^ ^]^ Once the protein is recognized, Hsc70 interacts with the lysosomal receptor LAMP‐2A. This interaction causes LAMP‐2A monomers to oligomerize and form a translocation channel, through which the targeted proteins are delivered into the lysosome with the assistance of Hsc70, which assists by pulling the unfolded protein across the lysosomal membrane.^[^ ^42^, ^43^ ^]^ Unlike macroautophagy and microautophagy, which degrade larger cytoplasmic contents (e.g., whole organelles), CMA is highly specific and tightly regulated by LAMP‐2A availability and expression.

### 2.2 Autophagy and Other Cell Death Mechanisms

Although autophagy is often considered a survival mechanism, it can lead to autophagy‐dependent cell death (ADCD) or exacerbate other cell death pathways when activated excessively or dysregulated. Autophagy is closely linked to apoptosis, considered the most central mechanism of cell death, and plays a vital role in regulating other cell death mechanisms, including necrosis, necroptosis, ferroptosis, and pyroptosis.^[^ ^44^, ^45^ ^]^

The roles of autophagy in cell death can be classified into ADCD (or autophagic cell death, ACD) and autophagy‐mediated cell death (AMCD).^[^ ^44^ ^]^ ACD depends strictly on the autophagy machinery and is independent of other programmed death. ACD includes ER‐phagy, mitophagy, and autosis. In AMCD, autophagy is not the primary cause of death but acts as a meditator or regulator of other modes of cell death (i.e., apoptosis, necroptosis, and ferroptosis). Both ACD and AMCD can be dependent on each other, triggered simultaneously, and may switch between the two.^[^ ^44^ ^]^ For instance, autophagy can delay cell death by mitigating stress and promoting apoptosis through interactions with apoptotic regulators like p53 or caspases.^[^ ^46^ ^]^ Autophagy can prevent necrosis and necroptosis by maintaining energy levels but may contribute to necrotic‐like outcomes under extreme stress.^[^ ^44^ ^]^ Ferroptosis can be facilitated by autophagy through ferritinophagy and lipophagy, which increase iron availability and lipid peroxidation.^[^ ^47^ ^]^ Moreover, autophagy modulates pyroptosis by clearing inflammasomes and damaged organelles but can also exacerbate inflammatory responses when dysregulated.^[^ ^48^ ^]^ Autophagy also interacts with less common forms of cell death such as parthanatos, NETosis (a neutrophil‐specific form of cell death), mitotic catastrophe, entosis (cell engulfment by neighboring cells), and methuosis (vacuole‐mediated nonapoptotic death).^[^ ^49^, ^50^, ^51^, ^52^ ^]^ Overall, autophagy plays an important role in cell fate decisions across various cell death modalities depending on the cellular context and stress conditions.

### 2.3 Autophagy and Cancer

**Figure 2:**
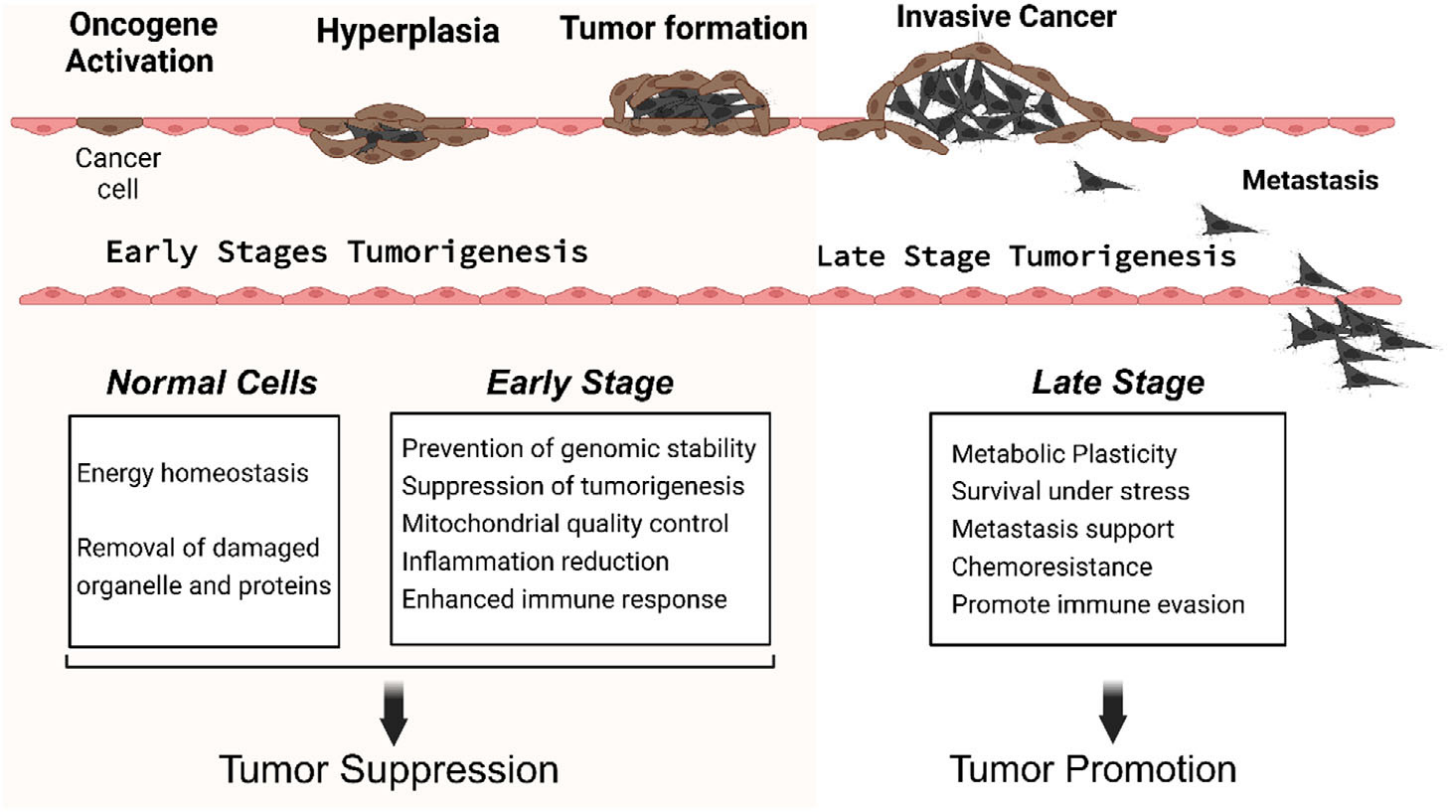
Dual effect of autophagy both as a tumor suppressor and promoter. The balance between tumor suppression and promotion shifts during cancer progression.

Autophagy plays a complex, context‐dependent role in tumor development, maintenance, and progression, acting as both a promoter and a suppressor depending on TME and the stage of cancer development (**Figure** **2**).^[^ ^53^, ^54^, ^55^, ^56^ ^]^ In the early stages, autophagy functions as a tumor suppressor by regulating cellular homeostasis through the degradation of damaged proteins and organelles, reducing overall genomic instability.^[^ ^55^, ^57^ ^]^ This process helps eliminate potentially tumorigenic cells and limits inflammation, which can contribute to tumor initiation. However, as tumors progress, autophagy is hijacked to promote cancer cell survival by providing metabolic plasticity to tumor cells, allowing them to survive under stressful conditions such as nutrient deprivation and hypoxia often faced by solid tumors.^[^ ^58^, ^59^, ^60^ ^]^ Autophagy supports metastasis by helping tumor cells evade cell death during detachment from the extracellular matrix.^[^ ^59^ ^]^ Additionally, autophagy contributes to immune evasion and chemoresistance by protecting tumor cells against treatment‐induced apoptosis and promoting tumor dormancy.^[^ ^57^, ^61^ ^]^ Understanding the interplay and function of autophagy is crucial for developing effective cancer therapies, as inhibiting prosurvival autophagy has been shown to enhance tumor cell death and trigger apoptosis in preclinical models.^[^ ^62^, ^63^ ^]^

#### 2.3.1 Autophagy in Tumor Promotion

Autophagy‐driven tumor progression relies on several pathways that create conditions favorable for tumor growth, such as hypoxia, nutrient starvation, immune evasion, and chemotherapy resistance.

##### Hypoxia

Within the tumor microenvironment (TME), cells are exposed to low oxygen levels resulting from the rapid and abnormal growth of tumors, affecting their ability to maintain sufficient blood flow. Under hypoxic conditions, autophagy is activated through hypoxia‐inducible factors (HIFs), particularly the HIF‐1α/AMPK pathway. This promotes cell survival by degrading damaged mitochondria and preventing the accumulation of reactive oxygen species (ROS).^[^ ^64^ ^]^ Additionally, hypoxia impairs cytotoxic T cell function and promotes the recruitment of regulatory T cells, lowering tumor immunogenicity.^[^ ^40^ ^]^ The activation of HIF1A triggers signaling cascades that impair nitric oxide signaling, leading to the shedding of MHC class I chain‐associated molecules. This ultimately disrupts immune surveillance by natural killer cells.^[^ ^65^ ^]^ HIF1A also induces target genes such as PD‐L1 and CCL28, promoting immune escape and tumor regrowth.^[^ ^66^, ^67^ ^]^ Hypoxia is also associated with resistance to chemotherapy, as observed with doxorubicin (DOX) in human breast cancer^[^ ^68^ ^]^ and *cis*‐diamine, dichloro platinum II (CDDP), and 3‐bis, 2‐chloroethyl‐1‐nitrosurea (BCNU) in brain glioma cells.^[^ ^69^ ^]^

##### Immune Evasion

The induction of autophagy in immune cells such as macrophages and dendritic cells influences antigen presentation and T cell activity, further aiding tumor immune evasion.^[^ ^70^, ^71^, ^72^ ^]^ Furthermore, deletion of the autophagy gene ATG5 in tumor‐associated macrophages (TAMs) increased the expression of immunosuppressive markers and promoted diethylnitrosamine (DEN)‐induced hepatocarcinogenesis.^[^ ^73^ ^]^

Autophagy may promote M2 macrophage polarization, which is generally considered anti‐inflammatory and promotes tumorigenesis.

##### Drug Resistance

Drug resistance remains a significant challenge in oncology, limiting the effectiveness of cancer therapies. Several factors contribute to drug resistance, including drug sequestration, metabolic reprogramming, genetic instability, TME modulation, and immune evasion.^[^ ^62^ ^]^ Autophagy is reported to be the primary mechanism behind acquired resistance to several drugs, such as paclitaxel (PTX), oxaliplatin, and cisplatin.^[^ ^74^, ^75^ ^]^ Autophagy was upregulated following cisplatin treatment to mitigate cisplatin‐induced kidney injuries. In this context, administering chloroquine (CQ) would exacerbate this injury.^[^ ^76^, ^77^ ^]^ Drug sequestration occurs when therapeutic agents are trapped within intracellular compartments, such as lysosomes or autophagosomes, preventing the drugs from reaching their intended targets and diminishing their cytotoxic effects. PTX, a mitotic inhibitor used in aggressive cancers, induces autophagy, forming acidic vesicular organelles that protect cancer cells from stress and apoptosis.^[^ ^78^, ^79^ ^]^ Combining chemotherapy with autophagy inhibitors can overcome drug sequestration and resensitize cancer cells.^[^ ^62^, ^79^ ^]^ Autophagy has also been shown to activate the expression of the multidrug‐resistant (MDR)1 gene and drug efflux pumps in lung adenocarcinoma A549 cells, enhancing cancer cell survival in response to drugs such as Adriamycin.^[^ ^80^ ^]^ Upregulation of ATG‐5 and ATG‐10, key regulators of protective autophagy, is associated with chemoresistance in gastric and colorectal cancer.^[^ ^81^, ^82^, ^83^ ^]^

##### Metabolism

Autophagy‐driven metabolic reprogramming and genetic instability contribute to therapeutic resistance.^[^ ^84^ ^]^ By recycling metabolites and maintaining energy production, tumor cells can withstand chemotherapy and adapt to stress. The downregulation of Beclin‐1 has been linked to the environmental adaptation of cancer cells, allowing them to reprogram glucose metabolism and proliferate; however, it can also be upregulated depending on tumor type.^[^ ^85^, ^86^, ^87^ ^]^

#### 2.3.2 Autophagy in Tumor Suppression

Autophagy is critical in inhibiting tumorigenesis by reducing inflammation, maintaining genome stability, and enhancing immune responses. The degradation of damaged organelles, proteins, and foreign antigens helps prevent harm to healthy cells and reduces cancer risk.^[^ ^88^, ^89^ ^]^ Autophagy suppresses chronic inflammation by clearing damaged molecules that might trigger immune responses, preventing a protumorigenic environment.^[^ ^90^ ^]^ It eliminates toxic mutagens and prevents the accumulation of genetic defects.^[^ ^91^ ^]^ Several tumor suppressor genes, such as PTEN and LKB1, are involved in activating autophagy pathways, further supporting its role in tumor suppression.^[^ ^90^ ^]^ Autophagy adaptor protein p62 (SQSTM1) regulates autophagy and inflammation by ubiquitinating proteins, including itself, for degradation. High levels of p62, often observed in cancers, can lead to excessive activation of NF‐κB, promoting tumor development. Therefore, clearing excess p62 through autophagy helps prevent tumorigenesis linked to inflammation.^[^ ^36^ ^]^

Autophagy enhances both innate and acquired immune responses. It stimulates antitumor immunity by promoting cytokine release in antigen‐presenting cells, boosting antigen presentation of MHCI and MHCII molecules, and supporting T‐cell survival.^[^ ^92^, ^93^ ^]^ The immune system is closely connected to the mTOR pathway, which regulates cellular metabolism, autophagy, and apoptosis.^[^ ^94^, ^95^, ^96^ ^]^ mTORC1, a key regulator of cell growth and metabolism, inhibits autophagy by phosphorylating ULK1/2 and the VPS34 complex, suppressing their activity. Additionally, mTORC1 blocks the expression of lysosomal and autophagy‐related genes through phosphorylation of the transcription factor TFEB, further inhibiting autophagy.^[^ ^97^ ^]^ By codelivering autophagy inducers, autophagy can be pushed beyond a critical threshold, overriding the protective effect in tumor cells and triggering ACD, thus enhancing therapeutic efficacy.^[^ ^98^ ^]^

#### 2.3.3 Autophagy and Tumor Subtypes

The role of autophagy significantly varies across different cancer types and their subtypes, highlighting its nature and role within different tumor types. In nonsmall cell lung cancer (NSCLC), autophagy‐related tumor subtypes exhibit distinct gene expression profiles, prognoses, mutation signatures, and immune infiltration patterns. A study identified 23 prognostic autophagy‐related genes that were differentially expressed, including seven downregulated and 16 upregulated genes, allowing the classification of NSCLC patients into two groups: Signature A (23 genes) and Signature B (12 genes).^[^ ^99^ ^]^ Group B was associated with a worse prognosis and increased expression of genes involved in cell proliferation and immune checkpoint signaling.^[^ ^100^ ^]^ Breast cancer molecular subtypes also show varying sensitivity to autophagy inhibition, with triple‐negative breast cancer (TNBC) being the most sensitive.^[^ ^101^, ^102^ ^]^ TNBC specifically expresses ATG5,^[^ ^101^ ^]^ promoting cell migration, while ER‐positive breast cancers are associated with BECN1 expression, inhibiting overall migration.^[^ ^103^ ^]^ Pancreatic ductal adenocarcinoma (PDAC) is another cancer subtype that displays a complex relationship with autophagy, where the loss of ATG5 increases premalignant lesion formation but inhibits progression to invasive PDAC.^[^ ^104^, ^105^ ^]^ Whereas in established PDAC tumors, autophagy supports glucose metabolism and tumor growth. Endocrine‐dependent cancers also exhibit complex and independent autophagy roles in cancer initiation, tumorigenesis, metastasis, and treatment response.^[^ ^58^ ^]^ These variations highlight the context‐dependent nature of autophagy in cancer and underscore the importance of considering specific tumor types and subtypes when developing autophagy‐targeting therapies.

## 3 Nanomedicine for Autophagy Modulation

Conventional cancer therapeutics often encounter challenges that limit their efficacy, including rapid drug clearance, low tumor accumulation, poor water solubility, instability, off‐site toxicity, immunogenicity, and drug resistance.^[^ ^106^ ^]^ These challenges can be overcome by leveraging nanoscale drug delivery to enhance drug stability, improve solubility, and prolong circulation time. Most NPs benefit from passive targeting through enhanced permeability and retention (EPR). Still, they can also be used for active targeting by functionalizing their surface with ligands and biomimics to bind to specific tumor receptors, overcome certain biological barriers, and improve tumor penetration. Moreover, NPs offer the possibility of controlled and stimuli‐responsive drug release at tumor sites, improving therapeutic precision and reducing system toxicity. The delivery of multiple therapeutic agents with distinct mechanisms of action and diverse chemical natures (small drug molecules and biologics such as antibodies, peptides, enzymes, and nucleic acids) can significantly enhance the therapeutic outcomes of single therapies but also permits the integration of multiple therapeutic modalities, such as immunotherapy, chemotherapy, gene therapy, and imaging, within a single platform.

These tools have been exploited to enhance the effectiveness of autophagy‐targeting therapies, particularly for drug combinations, as most current autophagy modulation strategies are added to enhance the efficacy of other drugs (except mTOR inhibitors, which are used as primary therapeutic agents). Autophagy‐modulating drugs can be administered as single agents and, more often, in combination with other therapeutics either separately or physically coloaded onto the same nanoplatforms. Another particularity of using nanomedicine for autophagy modulation is that beyond their role as drug carriers, several nanomaterials can modulate autophagy on their own (see Section 3.1), broadening their potential therapeutic applications. Examples of novelties in the design of nanomedicine for autophagy modulations include nanoreactors (NRs) that allow directing oncoproteins for autophagic degradation,^[^ ^107^ ^]^ the inclusion of chemical motifs sensitive to autophagy enzymes toward on‐demand autophagy triggered specific release,^[^ ^108^ ^]^ designing lysosomal‐targeting aggregated NPs (LTANP),^[^ ^109^ ^]^ and the modification of existing autophagy inhibitors, such as hydroxychloroquine, to allow self‐assembly, enhancing, thus, their potency and therapeutic efficacy and alleviating the need for further formulation optimizations.^[^ ^110^, ^111^ ^]^ These examples and others are covered in the following sections. **Table** **1** summarizes specific strategies for improving drug delivery and efficacy of autophagy modulators.

**Table 1:** The rationale for using NPs to deliver autophagy modulators in cancer therapy.

Rationalea)	Example(s)/Mechanism	References
Overcome poor water solubility	Nab‐sirolimus (autophagy inducer) is formulated to overcome sirolimus's poor water solubility by binding to albumin. It also improves tumor accumulation and reduces systemic side effects.	[456]
Prolong blood circulation	DMA‐modified PLGA NPs size (122 nm) and charge‐neutral surface (0.21 mV) enabled long blood circulation.	[292]
Overcoming biological barriers	Fe‐CDs decorated with angiopep‐2 (Fe‐CDs@Ang) enhanced BBB transcytosis and accumulation in brain tissue parenchyma in GBM mouse models.	[269]
Enhance tumor targeting and accumulation	Passive targeting	
	BAQ NPs accumulate in mouse models of pancreatic (MIA PaCa‐2) and colon (HT29) tumors	[110]
	Active targeting	
	Antibody Cetuximab‐decorated Ag2S QDs (mPEG–Ag2S–Cet) improved targeting specificity towards EGFR‐overexpressing cells.	[239]
	Stimuli‐responsive drug release PLGLAG peptide is cleaved by overexpressing matrix metalloproteinase‐2 (MMP‐2) in TME Disulfide‐bonded NPs release epirubicin (EPI) and STF62247 in high‐GSH environments in TME.	[457]
	Biomimicry PLGA coated with TRAIL membrane vesicles (TH‐NP) loaded with HCQ and OXA accumulate in the tumor site through TRAIL binding to DR4/5 on HCC cells.	[275]
	On‐demand autophagy‐triggered release NPs trigger mild autophagy after OXA release, thus activating ATG4‐mediated TFG cleavage inside cells to release STF‐62247.	[108]
Enhance tumor penetration	DMA‐modified PLGA NPs expose cationic GR9 peptides under acidic conditions for better penetration into melanoma tumors.	[292]
Synergistic combination therapies	Autophagy inhibition by LY294002 improved the sensitivity of cancer cells to 5‐FU	[458]
	Polymer complex (PEI‐oleic acid) co‐delivers atezolizumab, paclitaxel, ovalbumin (OVA), CpG, and CQ, enhancing the effectiveness of both chemotherapy and immunotherapy	[297]
Novel mechanism of action	NRs utilize a mutp53‐binding peptide (MBP) for specific recognition and a cationic lipid (DOTAP) to induce autophagosome formation, facilitating the targeted autophagic degradation of mutp53. This enhances therapeutic efficacy in p53‐mutated cancer cells.	[107]
	LTANP aggregate at low pH within the lysosomes causing LMP.	[109]
Self‐assembling autophagy modulator	BAQ derivatives self‐assemble into NPs, accumulate at the tumor site and have significant antitumor efficacy as single agents and in combination therapy.	[110, 111]
Diagnostic and monitoring	Pt(IV)/CQ/PFH‐DPPA‐1 NPs enable US imaging through perfluoro hexane (PFH) while delivering CQ to inhibit protective autophagy caused by cisplatin in breast cancer	[459]
Intrinsic autophagy modulators	IONPs, AuNPs, AgNPs, etc.	Section 3.1
Multimodal therapies	PBC, a conjugate of pheophorbide A and BAQ, self‐assembles into NPs. PBC has autophagy inhibition properties and is used for PDT, PTT, and imaging.	[111]
Reduction of off‐target toxicity	NPs can reduce drug toxicity by improving tumor targeting and accumulation, controlling drug release to avoid peak concentrations, and reducing local side effects at the injection site.	

a) BBB, blood‐brain barrier; CQ, chloroquine; GBM, Glioblastoma; HCC, hepatocellular carcinoma; IONPs, iron oxide nanoparticles; LMP, lysosomal membrane permeabilization; OXA, oxaliplatin; PEI, polyethyleneimine; PFH, perfluorohexane; PLGA, poly(lactic*‐co*‐glycolic acid); PTT, photothermal therapy; PDT, photodynamic therapy; 5‐FU, 5‐fluorouracil; TME, tumor microenvironment; TRAIL, TNF‐related apoptosis‐inducing ligand.

**Figure 3:**
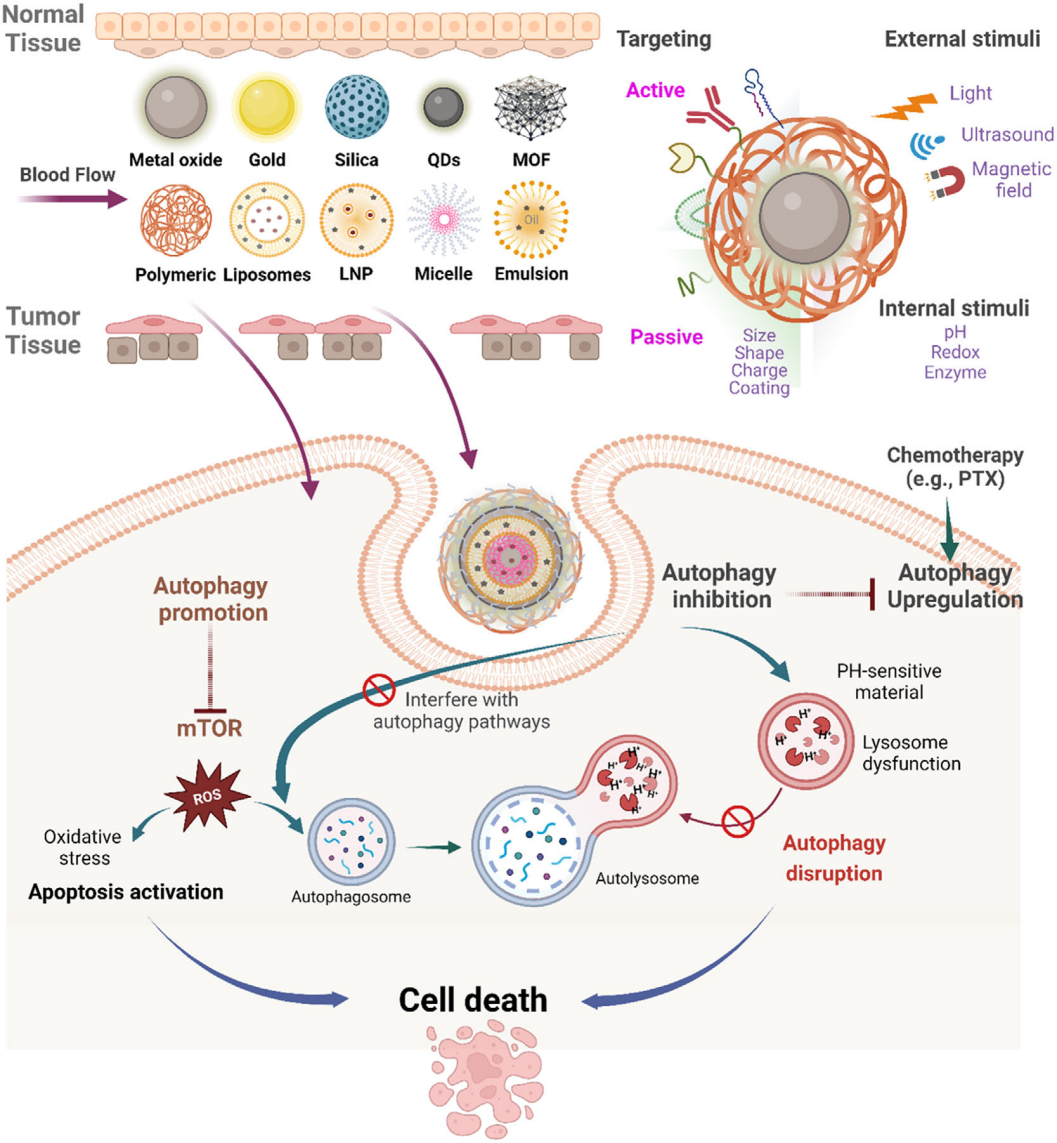
Nanomedicine for autophagy modulation in cancer therapy utilizes NPs to deliver anticancer therapies via passive targeting (EPR effect: size, charge, shape) and active targeting (ligands, antibodies, stimuli–responsive materials, biomimics). Tumor‐specific delivery achieves effective cell killing by modulating autophagy—either through inhibition or promotion—alone or in combination with other treatments. Conventional anticancer treatments often induce cytoprotective autophagy, making autophagy inhibition a synergistic strategy to enhance their efficacy. Alternatively, inducing excessive autophagy can promote ACD, offering a potential alternative therapeutic approach. A detailed scheme for inhibition mechanisms for inorganic and organic nanomaterials is found in Chen et al.^[^ ^391^ ^]^ Created in BioRender. Mahri, S. (2024) https://BioRender.com/a02t193.

Most NPs for autophagy modulation are administered intravenously and once in the bloodstream, NP size, charge, and surface chemistry, and to a lesser extent, shape, and elasticity affect their biodistribution, cellular uptake, interaction with the immune system and other biological systems (**Figure** **3**). Among these properties, surface properties influence the adsorption of proteins onto the NPs, forming a “protein corona.” The corona's composition affects NP biodistribution, cellular uptake, and interaction with the immune system. For instance, by modifying the composition of a single lipid (the SORT molecule) in lipid NPs (LNPs), their delivery could be skewed to specific organs such as the liver, spleen, or lungs. This effect is mainly attributed to the nature of protein corona on these LNPs.^[^ ^112^ ^]^ Besides passive targeting, active targeting (e.g., antibodies, peptides, biomimics) can guide NPs to specific organs/tissues and across various biological barriers. In the TME, NPs can release their cargo or be taken up by tumor cells. Stimuli such as low pH, glutathione (GSH), high ROS, hypoxia, or tumor‐specific enzymes can trigger drug release by breaking down NPs coatings or linkers, enabling localized delivery.^[^ ^113^, ^114^ ^]^ Once NPs reach tumor cells, they typically enter via endocytosis, including clathrin‐dependent, caveolin‐dependent, macropinocytosis, or phagocytosis pathways.^[^ ^115^, ^116^ ^]^ Inside the cell, NPs are often confined in endosomes or lysosomes and usually need to escape into the cytoplasm to effectively release their therapeutic payload. This process may involve endosomal escape mechanisms like membrane disruption or pH‐triggered destabilization. Avoiding endosomal escape is sometimes desired and is used by some autophagy modulators.^[^ ^115^ ^]^

NP characteristics, including size, surface properties, and chemical composition, can also impact autophagic modulating properties by influencing interaction with cellular membranes, organelles, intracellular signaling pathways, cell uptake, intracellular localization, and toxicity, ultimately affecting autophagy regulation. Examples of these interactions are provided in **Table** **2**.

**Table 2:** Examples of the influence of NP properties on autophagy.

NPs property	Impact/mechanism	Example	References
Size	Size may affect cell uptake and crossing biological barriers, causing intracellular/lysosomal accumulation and interference with autophagic flux.	Smaller AuNPs had more efficient cell uptake, lysosome alkalinization, and autophagosome‐lysosome fusion blockage. (10 nm > 25 nm > 50 nm)	[149]
		Gold nanopyramids coated with titanium dioxide (NBP/TiO_2_) induced autophagosome accumulation in U‐87 MG; this effect was more pronounced in smaller NBP/TiO2 structures (47 nm > 95 nm > 142 nm).	[163]
Surface coating	Surface coating may affect protein adsorption, cell uptake pathway, and intracellular trafficking.	Gold nanopyramids coated with titanium dioxide (NBP/TiO_2_) induced a more significant autophagosome accumulation in U‐87 MG cells compared to bare, PEG, or silica‐coated NPs	[163]
Dispersity	Aggregated NPs tend to adhere to cell surfaces more effectively, leading to increased endocytosis (cellular uptake)	Aggregated IONPs induced a significant autophagic effect in comparison with well‐dispersed NPs.	[126]
		Stabilized IONPs through surface coatings (dopamine (DA) or DOPAC) were less likely to aggregate and decreased the autophagic response.	[460]
Protein corona	Protein corona (influenced by surface composition and charge) influences interaction with cells and intracellular targets.	Protein corona on AuNP affected trafficking through cellular compartments, which affected autophagy via CMA.	[461]

### 3.1 Nanomaterial with Intrinsic Autophagy‐Modulating Properties

Various nanomaterials have been reported to intrinsically modulate autophagy in normal and cancer cells.^[^ ^117^ ^]^ They have been used as standalone agents and as carriers to deliver other therapeutic cargos. These include metal oxides (e.g., iron oxide), gold NPs (AuNPs), silver NPs (AgNPs), silica NPs, quantum dots (QDs), nanosized carbon‐based nanomaterials (e.g., carbon nanotubes, graphene, carbon dots, fullerene), metal–organic frameworks (MOFs), and some polymers. These nanomaterials can modulate autophagy through various pathways, often with nonspecific effects. They may either induce or inhibit autophagy and, in some cases, exhibit dual effects depending on the biological system and the specific modifications of the nanomaterial.

#### 3.1.1 Inorganic Nanomaterials

##### Metal‐Based Nanomaterials

###### Iron Oxide Nanomaterials

Iron oxide NPs (IONPs) are versatile materials with numerous biomedical applications in imaging, diagnostics, and therapeutics.^[^ ^118^, ^119^, ^120^, ^121^ ^]^ Among NPs in clinical trials, 11% are metal based, predominantly represented by iron preparations.^[^ ^122^ ^]^ IONPs were among the first NPs adopted clinically for their safety and biocompatibility. They have received FDA approval for gastrointestinal imaging, MRI, and managing iron deficiency anemia. Notably, NanoTherm, an aminosilane‐coated IONP, is approved for intratumoral injection for glioblastoma thermal ablation and is currently being investigated for prostate cancer (NCT05010759).^[^ ^118^ ^]^ Preclinically, cancer therapy strategies using IONPs focus on magnetic hyperthermia, tumor sensitization to chemotherapy and radiotherapy, and carriers for drug delivery.^[^ ^123^, ^124^, ^125^ ^]^

IONPs’ autophagy‐modulating properties have been shown in a plethora of cell lines, including HeLa,^[^ ^126^ ^]^ OPM2,^[^ ^127^ ^]^ A549,^[^ ^128^ ^]^ MCF‐7,^[^ ^129^ ^]^ RAW264.7,^[^ ^130^ ^]^ monocytes,^[^ ^131^ ^]^ SKOV‐3,^[^ ^132^ ^]^ OECM1,^[^ ^133^ ^]^ HepG2,^[^ ^134^ ^]^ cerebral endothelial cells,^[^ ^135^ ^]^ PC12,^[^ ^136^ ^]^ U2OS,^[^ ^137^ ^]^ and dendritic cells.^[^ ^138^ ^]^ IONPs have shown promise as anticancer agents by selectively inducing prodeath autophagy in cancer cells primarily through excessive generation of ROS.^[^ ^128^, ^136^ ^]^ Nonetheless, a prosurvival autophagy effect was reported in human blood cells by attenuating cell death induced by bortezomib and DOX.^[^ ^127^ ^]^ Key signaling pathways involved in IONP‐induced autophagy include the AMPK‐mTOR‐AKT pathway, toll‐like receptor‐4, Beclin‐1/Bcl‐2/VPS34 complex, Beclin‐1/ATG5, and Cav1‐Notch1/HES1.^[^ ^127^, ^128^, ^130^, ^139^, ^140^ ^]^ Jin et al. demonstrated that superparamagnetic iron oxide NPs (SPIONs), Resovist and Feraheme, can induce autophagy both in vivo in mice liver and in vitro in macrophages by interacting with TLR4 and triggering a signaling pathway independent of the classic p62 reduction pathway.^[^ ^130^ ^]^ Chemical composition, surface modification, and particle size and charge affect the toxicity and autophagy‐modulating properties of IONPs.^[^ ^126^, ^132^, ^136^, ^141^ ^]^ This is particularly important for IONPs which are often coated with dextran, PEG, pluronic, citrate, and aminosilane to enhance their colloidal stability.^[^ ^126^, ^132^ ^]^

**Figure 4:**
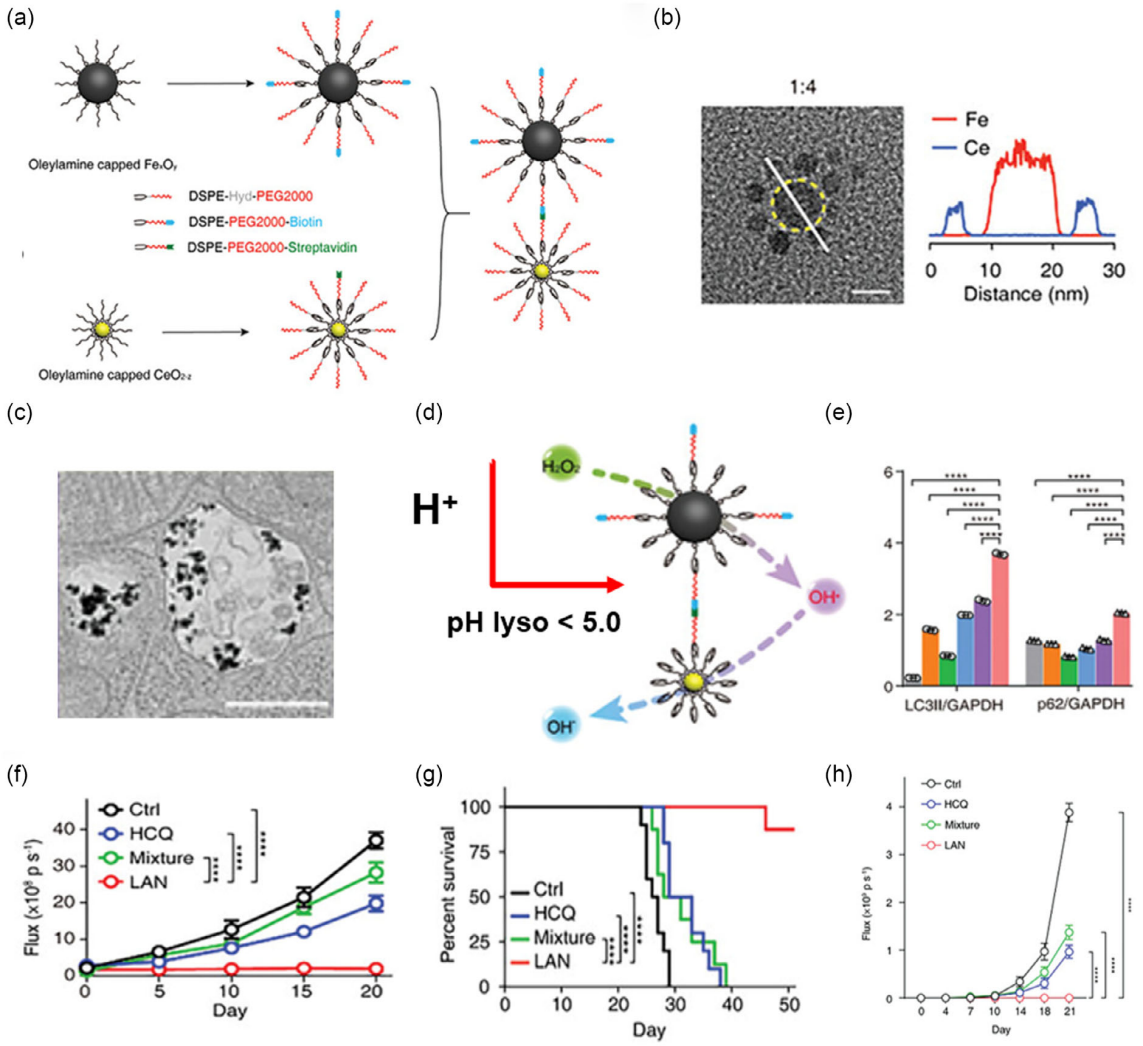
a) LAN synthesis involves conjugating biotin‐modified FexOy NPs with streptavidin‐modified CeO_2−*z*_ NPs. Both NPs contain pH‐labile PEGs; b) transmission electron microscopy (TEM) images of LAN with a 1:4 [Fe]:[Ce] ratio. Scale bars, 10 nm; (left) energy‐dispersive X‐ray spectroscopy elemental line scan of the white solid line in LAN (right). c) Bio‐TEM images show LAN accumulation inside lysosomes of LLC cells. d) At acidic pH (H+) of lysosomes, LAN undergoes a cascade of catalytic reactions that neutralize the lysosome. e) Western blot analysis quantification of LC3‐I/II and p62 levels, confirming autophagy inhibition in LLC. f) Bioluminescence flux measuring tumor growth in orthotopic LLC model mice. g) Survival curves showing LAN treatment prolonged animal survival. h) Quantification of the growth of metastatic LLC cancers. Reproduced with permission.^[^ ^143^ ^]^ Copyright 2024, Wiley.

IONPs are utilized alone and in combination with other chemotherapeutic drugs, including other autophagy modulators. Chen et al. showed that combining with PTX (IONP@PTX) effectively inhibits tumor growth in glioblastoma (GBM) xenograft mice by enhancing the autophagy‐dependent ferroptosis pathway.^[^ ^142^ ^]^ Notably, the efficacy of this combination was reduced by 3‐MA and amplified by rapamycin. Recently, Pan et al. developed nanoclusters of FexOy and CeO_2−*z*_, wherein biotinylated FexOy NPs were combined with streptavidin‐coated CeO_2−*z*_ NPs to obtain LAN in which FexOy core is coated by CeO_2−*z*_ satellites (**Figure** **4**).^[^ ^143^ ^]^ Both NPs were modified with polyethylene glycol (PEG) (DSPE‐Hydazone‐PEG2000) to prolong their blood circulation and improve stability. In the acidic environment of lysosomes, hydrazone bonds are cleaved, stripping LAN from their protective PEG shell. Fe_*x*_O_*y*_ NPs convert H_2_O_2_ into •OH, which is then captured and converted into hydroxide ions by CeO_2−*z*_ NPs, leading to lysosomes alkalinization and, thus, inhibiting autophagic flux and inducing apoptosis in cancer cells. LANs were shown to accumulate in the tumors 24 h post‐injection and effectively inhibit both local and systemic tumor growth and metastasis in LLC orthotopic mouse models. LANs have demonstrated superior efficacy compared with HCQ alone or a simple mixture of Fe_*x*_O_*y*_ and CeO_2−*z*_ with minimal off‐tumor toxicities.

###### Gold Nanoparticle

Gold NPs (AuNPs) are widely investigated in drug delivery due to their unique properties such as stability, biocompatibility, size tunability, low toxicity, and ease of modification.^[^ ^144^, ^145^ ^]^ They are also utilized in imaging, phototherapy, radiosensitization, and biosensing.^[^ ^146^, ^147^, ^148^ ^]^ Despite extensive research, only a few gold‐based nanomedicines are in clinical trials, and none have received FDA approval.^[^ ^147^ ^]^

AuNPs have also been reported to induce autophagy in general.^[^ ^48^, ^49^ ^]^ but also to block the autophagic pathway by accumulating in the lysosomes and impairing their function.^[^ ^149^ ^]^ Factors such as surface chemistry, particle size, and dispersity have been reported to influence the autophagy‐modulating properties of AuNPs.^[^ ^126^, ^149^, ^150^ ^]^ Proposed mechanisms of autophagy dysregulation include ROS generation, mitochondrial damage, and impairment of lysosome function.^[^ ^151^, ^152^ ^]^ AuNPs have been reported to enter cells through size‐dependent endocytosis and impair lysosomal function through alkalinizing lysosomal pH, leading to autophagosome accumulation.^[^ ^149^, ^153^ ^]^ Despite having the same particle volume, AuNPs nanospheres (20 nm) were taken up more efficiently compared to nanorods (40 nm). AuNPs can be easily modified to deliver antibodies (e.g., anti‐HER2 anti‐EGFR), nucleic acids, chemotherapeutics, and other autophagy modulators such as ABN‐AZD, an albumin‐stabilized gold nanocluster with AZD8055.^[^ ^154^, ^155^, ^156^, ^157^, ^158^, ^159^, ^160^ ^]^ Coating AuNPs with autophagy promoter Ziyuglycoside I (ZgI) enhanced their autophagy‐promoting effect in hematopoietic stem cells.^[^ ^161^ ^]^ Zgl II, the active metabolite of ZgI, induces autophagy by inhibiting the Akt/mTOR pathway, demonstrating significant antitumor activity against colorectal cancer (CRC) cells in vitro and in vivo.^[^ ^162^ ^]^

Wan et al. reported a novel autophagy inhibitor, gold nanopyramids coated with titanium dioxide (NBP/TiO2).^[^ ^163^ ^]^ NBP/TiO2 induced more autophagosome accumulation in U‐87 MG cells than bare, PEG, or silica‐coated NPs. Notably, this effect was more pronounced in smaller NBP/TiO2 structures (47 nm > 95 nm > 142 nm). Activation of the AMPK/mTOR pathway was reported to cause autophagosome accumulation by blocking autophagosome‐lysosome fusion. Additionally, autophagy inhibition by NBP/TiO2NPs enhanced the effects of bortezomib and photothermal therapy (PTT). In another study, PEG‐AuNPs inhibited tumor growth in mice‐bearing subcutaneous Hepa 1–6 cell tumors. Suppression of M2 polarization in TAMs via lysosomal dysfunction and inhibition of autophagic flux was reported to be key in PEG‐AuNPs’ efficacy.^[^ ^164^ ^]^ Additionally, AuNPs accentuated the TRAIL‐induced apoptosis in nonsmall‐cell lung cancer cells in vitro and in vivo through Drp1‐dependent mitochondrial fission and increased autophagy.^[^ ^165^ ^]^

###### Silver Nanoparticles

Silver NPs (AgNPs) have a long medical history as antimicrobial agents against bacteria, parasites, viruses, and fungi.^[^ ^166^, ^167^, ^168^, ^169^ ^]^ They have also been investigated for their antitumor properties.^[^ ^170^, ^171^, ^172^ ^]^ AgNPs‐induced autophagy has been demonstrated in multiple normal and cancer cell lines and animal models.^[^ ^173^, ^174^ ^]^ Notably, autophagy induction has been observed in PDAC (PANC‐1),^[^ ^175^ ^]^ liver cancer (HepG2),^[^ ^176^ ^]^ neuroblastoma (SH‐SY5Y),^[^ ^177^ ^]^ renal carcinoma (A498 and HEK293T),^[^ ^117^, ^178^ ^]^ lung epithelial cancer (A549),^[^ ^179^ ^]^ colorectal adenocarcinoma (HT‐29),^[^ ^180^ ^]^ breast cancer (SKBR3, MCF‐7, MDA‐MB‐468),^[^ ^181^, ^182^ ^]^ human prostate cancer (PC‐3),^[^ ^117^, ^178^ ^]^ HeLa cells,^[^ ^183^ ^]^ and monocytic (THP‐1).^[^ ^184^ ^]^

AgNPs autophagy induction is marked by an increase in autophagosome formation and the presence of autophagic vacuoles. AgNPs enter cells by phagocytic and endocytic pathways and are found within membrane‐bound structures such as intracellular vesicles and late endosomes.^[^ ^185^ ^]^ The acidic environment within lysosomes facilitates the release of Ag ions and AgNPs, which generate ROS that cause mitochondrial dysfunction, ER stress, DNA damage, autophagy, and apoptosis.^[^ ^186^ ^]^

While AgNPs exhibit intrinsic anticancer effects, most applications focus on utilizing them as carriers for chemotherapy or radiotherapy.^[^ ^186^ ^]^ Effective autophagy modulation has been reported with cisplatin, salinomycin, camptothecin, and MS‐275 (HDAC inhibitor).^[^ ^187^, ^188^, ^189^, ^190^ ^]^

AgNP‐induced autophagy in HepG2 cells was shown to be size dependent (10 nm > 50 nm > 100 nm) and was correlated with increased lysosomal activity and apoptosis.^[^ ^176^ ^]^ In HeLa cells, autophagy induction by AgNPs was cytoprotective, enhancing cell survival, while inhibiting autophagy (via Wortmannin or bafilomycin) increased AgNP cytotoxicity in B16 mouse melanoma and other cancer cell lines.^[^ ^183^ ^]^ Green synthesis of AgNPs has become popular recently. It relies on reducing silver ions (Ag+) to Ag0, which then forms AgNPs using plant or microbial extracts.^[^ ^191^ ^]^ For instance, Akter et al. reported that Ag‐NPs, produced by green synthesis using Brassica leaf aqueous extract, induced NF‐κB‐mediated autophagy in Caco‐2.^[^ ^192^ ^]^

###### Metal‐Organic Frameworks (MOFs)

MOFs are porous, crystalline materials composed of metal or metal ions (nodes) and organic ligands (linkers) linked by coordinative bonds.^[^ ^193^ ^]^ Common metal ions used in drug delivery applications include zirconium(IV), iron(III), and zinc(II), while ligands often feature multiple carboxyl or amine groups. Although generally rigid, MOFs exhibit some flexibility and have been effectively exploited to encapsulate small molecules, proteins, and nucleic acids.^[^ ^193^ ^]^ A recent study by Ge et al. used a zinc‐based zeolitic imidazolate framework (ZIF‐8) to develop a pH‐sensitive MOF nanocarrier coencapsulating curcumin and BMS1166, a PD‐1/PD‐L1 inhibitor.^[^ ^194^ ^]^ These pH‐sensitive NPs induced autophagy and decreased the intracellular pH, facilitating the release of curcumin and thereby amplifying the autophagic activity. The treatment resulted in a potent antitumor effect in a subcutaneous osteosarcoma (OS) model with improved long‐term immunity against tumor recurrence and enhanced dendritic cell maturation and tumor infiltration of CD8 + T lymphocytes. This strategy highlights the benefit of combining targeted autophagy modulation and immune checkpoint blockade in treating OS.^[^ ^194^ ^]^

Xiong et al. successfully loaded apoferritin with Cu(II) copper polypyridine (Aft‐Cu, 20 nm). This novel construct showed selective toxicity toward various cancer cell lines, including the drug‐resistant human colon adenocarcinoma cell line SW620/AD300, compared with noncancerous cell lines.^[^ ^195^ ^]^ Aft‐Cu exhibited a high cellular uptake and induced cell death via autophagy‐dependent apoptosis. Remarkably, Aft‐Cu showed strong tumor suppression in subcutaneous mouse model of MDR colon cancer, highlighting the potential of this therapeutic strategy in drug‐resistant tumors.

In addition to the autophagy‐modulating properties of some MOFs, they have also been used as carriers for other autophagy‐modulating drugs.^[^ ^196^, ^197^ ^]^ Wu et al. used Cu^2+^‐based MOFs grown on SiO2 nanosheets to load CQ. The system was modified with hyaluronic acid (HA) for improved CD44‐targeting. The resulting NPs were safe and effective, with good accumulation in the subcutaneous model of Hela tumor‐bearing mice. Once inside the cells, Cu^2+^ is reduced to Cu^+^ by GSH to generate hydroxyl radicals for ROS‐mediated chemodynamic therapy (CDT) (see Section 4.6). CQ is then released to inhibit autophagy, interfering with cells’ capability to defend against oxidative stress.^[^ ^197^ ^]^ In another study, an autophagy‐inhibitory MOF NR (CQ@ZIF‐GOx@C, ≈100 nm) was developed by coloading autophagy inhibitor CQ and glucose oxidase (GOx) followed by camouflaging the NPs with a cancer cell membrane^[^ ^196^ ^]^ Homotypic membrane‐camouflaged NPs had a good safety profile, better tumor accumulation, and tumor suppression than naked MOF NPs in the 4T1 tumor‐bearing mice model. This novel MOF construct works through several mechanisms. First, GOx consumes glucose, starving tumor cells and generating hydrogen peroxide (H_2_O_2_). The effect of GOx is enhanced by inhibiting autophagy CQ. Second, H_2_O_2_ induces oxidative stress, leading to immunogenic cell death (ICD) and macrophage polarization to the M1 phenotype. Other studies have developed MOFs that synergize with autophagy modulators such as CQ;^[^ ^198^ ^]^ however, these MOFs alone did not have autophagy‐modulating properties and were not designed to load these autophagy modulators and, therefore, fall outside the scope of this review.

Despite the potential of MOF as therapeutic agents and carriers for drug delivery, most research is still in the early preclinical phase. Their clinical translation faces general challenges common to inorganic NMs. These include concerns about safety, metal ion clearance, biodegradability, stability, synthesis, and large‐scale production, adding extra burden for their regulatory approval.

###### Other Metallic NPs

Other metallic and metalloid NPs were shown to modulate autophagy primarily through ROS generation and oxidative stress. They are effective as single agents but more consistently in combination with chemotherapy or radiotherapy, with promising results in several preclinical cancer models.^[^ ^199^ ^]^ These include zinc oxide,^[^ ^200^, ^201^, ^202^ ^]^ copper oxide,^[^ ^203^ ^]^ selenium,^[^ ^204^, ^205^, ^206^, ^207^, ^208^, ^209^, ^210^, ^211^ ^]^ titanium oxide,^[^ ^212^ ^]^ nickel,^[^ ^213^ ^]^ cobalt,^[^ ^214^ ^]^ vanadium pentoxide,^[^ ^215^ ^]^ cerium oxide,^[^ ^143^ ^]^ zirconium dioxide,^[^ ^216^ ^]^ and others. Like other metallic NPs, their clinical translation is hindered by the lack of thorough validation. Nonetheless, prioritizing physiologically relevant metals based on their natural abundance (e.g., zinc, copper, and selenium) could be a more strategic approach.

##### Silica Nanoparticles

Silica NPs (SiNPs), particularly mesoporous silica NPs (MSNs), are among the most used inorganic NPs for drug delivery due to their large surface area, tunable porosity, biocompatibility, stability, and ease of functionalization.^[^ ^217^, ^218^, ^219^ ^]^ SiNPs block autophagic flux and impair lysosomal function.^[^ ^220^, ^221^ ^]^ Other mechanisms implicated in SiNP‐induced cell death include oxidative stress, mitochondrial damage, ER stress, and apoptosis.^[^ ^222^, ^223^, ^224^, ^225^, ^226^ ^]^ Yang et al. showed that autophagy induction by MnO‐MS (mesopore‐encaged active MnOx in nanosilica) led to selective killing of lung cancer cells and suppression of tumor growth in vivo with minimal side effects.^[^ ^227^ ^]^ In another study, Predarska et al. demonstrated that MSNs loaded with platinum(IV) derivatives exhibit enhanced cytotoxicity across several human breast cancer cell lines compared with unloaded counterparts. Their toxicity was further improved in vitro by inhibiting autophagy using 3‐methyladenine (PI3K inhibitor) in 4T1. The authors did not assess whether this combination was effective in reducing tumor growth in the 4T1 orthotopic model of breast cancer in Balb/c mice.^[^ ^228^ ^]^ Zhan et al. reported autophagy‐inducing properties of RGD‐coated MSNs loaded with temozolomide and CQ (TMZ/CQ@MSN‐RGD) in U87 glioma cells.^[^ ^229^ ^]^ The combination with CQ helped reduce the autophagy induced by TMZ@MSN‐RGD and significantly increased apoptosis. Nonetheless, the authors did not investigate the role of bare MSN in autophagy, considering that TMZ is known to induce autophagy.^[^ ^230^, ^231^ ^]^

##### Quantum Dots (QDs)

QDs are semiconductor nanocrystals (e.g., CdSe, CdTe, ZnS, InP) with unique optical properties such as high photoluminescence efficiency, narrow emission spectra, and photostability.^[^ ^232^ ^]^ They have been used in drug delivery, bioimaging, PTT, and sensing.^[^ ^233^, ^234^ ^]^ Several QDs have been reported to modulate autophagy, such as CdTe,^[^ ^235^ ^]^ CdSeS@ZnS,^[^ ^236^ ^]^ CdTe/CdS 655 (QDs 655),^[^ ^237^ ^]^ realgar (As4S4),^[^ ^238^ ^]^ and Ag2S.^[^ ^239^ ^]^

For example, CdTe QDs induced apoptosis in normal liver cells (L02) and promoted protective autophagy in cancer liver cells (HepG2).^[^ ^235^ ^]^ Peynshaert et al. found that PEG‐coated QDs (CdSeS core and ZnS shell) cytotoxicity toward Hela cells was mediated by ROS production and impairment of lysosomal function leading to autophagy dysfunction. This toxicity was mitigated by coating QDs with 3‐mercaptopropionic.^[^ ^236^ ^]^ Upregulation of Beclin‐1 and LC3‐II was reported for Hederagenin‐loaded black phosphorus QDs (BPQDs) coated by platelet membranes in breast tumors.^[^ ^240^ ^]^ Cetuximab‐decorated Ag2S QDs (mPEG–Ag2S–Cet) enhanced 5‐FU efficacy by inhibiting 5‐FU‐induced cytoprotective autophagy in A549 cells.^[^ ^239^ ^]^ Furthermore, cetuximab improved targeting specificity toward EGFR‐overexpressing cells.

#### 3.1.2 Carbon‐Based Nanomaterials

Carbon nanomaterials, including graphene, carbon nanotubes, nanodiamonds, fullerenes, and carbon dots, offer good mechanical and chemical stability, high surface area, and easy surface functionalization, making them attractive for drug delivery.^[^ ^241^, ^242^, ^243^ ^]^ Graphene oxide (GO), in particular, has been shown to induce autophagy in various tumor cell lines.^[^ ^244^, ^245^, ^246^ ^]^ It has been successfully utilized to deliver other chemotherapeutics, including autophagy inhibitors like CQ and miR‐10.^[^ ^242^, ^247^, ^248^, ^249^ ^]^ They are also used in hybrid nanocomposites with gold and silver NPs.^[^ ^250^, ^251^ ^]^ GO can sensitize cancer cells to cisplatin by inducing early autophagy and promoting nuclear trafficking and necrosis.^[^ ^250^, ^252^, ^253^ ^]^ In colorectal tumors, GO autophagy modulation has been attributed to increasing ROS through the AMPK/mTOR/ULK1 pathway.^[^ ^254^ ^]^ Although not ye*t* tested in humans for cancer therapy, a first in human inhalation showed that inhaled GO nanosheets were well tolerated (NCT03659864).^[^ ^255^ ^]^

Carbon nanotubes and nanofibers, including multiwalled carbon nanotubes, acid‐functionalized single‐walled carbon nanotubes, and graphite carbon nanofibers, inhibit autophagy in various cell lines by generating ROS and disrupting lysosomal function, blocking autophagic flux.^[^ ^256^, ^257^, ^258^, ^259^ ^]^ However, these studies primarily focused on toxicological effects, with limited relevance to cancer therapy.

Carbon dots are carbon‐based QDs with high photoluminescence, chemical tuneability, biocompatibility, large surface area, and adjustable surface chemistry, making them suitable for drug delivery and theranostics.^[^ ^260^ ^]^ They are considered potential better alternatives to metal‐based QDs.^[^ ^245^ ^]^ Various carbon dots have been explored for cancer therapy.^[^ ^261^, ^262^ ^]^ N‐doped carbon dots triggered ROS‐mediated cytoprotective autophagy in Hepa1‐6 cells. However, autophagy was only evident at high doses (400 μg mL^−1^).^[^ ^245^ ^]^ Nitrogen–phosphorous‐doped carbon dots have excellent luminescence properties and are suitable for cell imaging. They induced cell cycle arrest, autophagy, and apoptosis in B16F10 melanoma cancer cells.^[^ ^263^ ^]^ This autophagy induction was marked by increased ATG5 and LC3‐II and decreased p62 expression. Furthermore, ROS generation disrupted mitochondrial function and led to high p21 expression.

Carbon dots’ size is tunable to the single‐digit nanometer range, facilitating their penetration into the blood–brain barrier (BBB), especially when decorated with ligands such as tryptophan, glucose, and large amino acid‐mimicking groups.^[^ ^264^, ^265^, ^266^, ^267^, ^268^ ^]^ Muhammad et al. designed ultrasmall carbon dots featuring single‐atom nanozymes (Fe‐CDs). Nanozymes are nanomaterials with intrinsic enzyme‐like properties that mimic enzymes.^[^ ^269^ ^]^ Fe‐N4 structure acts as an active enzyme center within the carbon matrix. Fe‐CDs mimic the activity of six enzymes, namely, OXD, POD, SOD, CAT, GPX, and TPx, recapitulating the intracellular antioxidant defense system for ROS‐mediated killing in drug‐resistant GBM cells. To enhance BBB transcytosis and accumulation in brain tissue parenchyma, Fe‐CDs were decorated with angiopep‐2 (Fe‐CDs@Ang), a synthetic LRP‐1 ligand. In GBM mouse models, Fe‐CDs@Ang inhibited tumor growth and demonstrated excellent safety, making them a promising, multifunctional platform for treating drug‐resistant GBM with minimal toxicity and high efficacy.

#### 3.1.3 Self‐Assembling Autophagy Inhibitor

**Figure 5:**
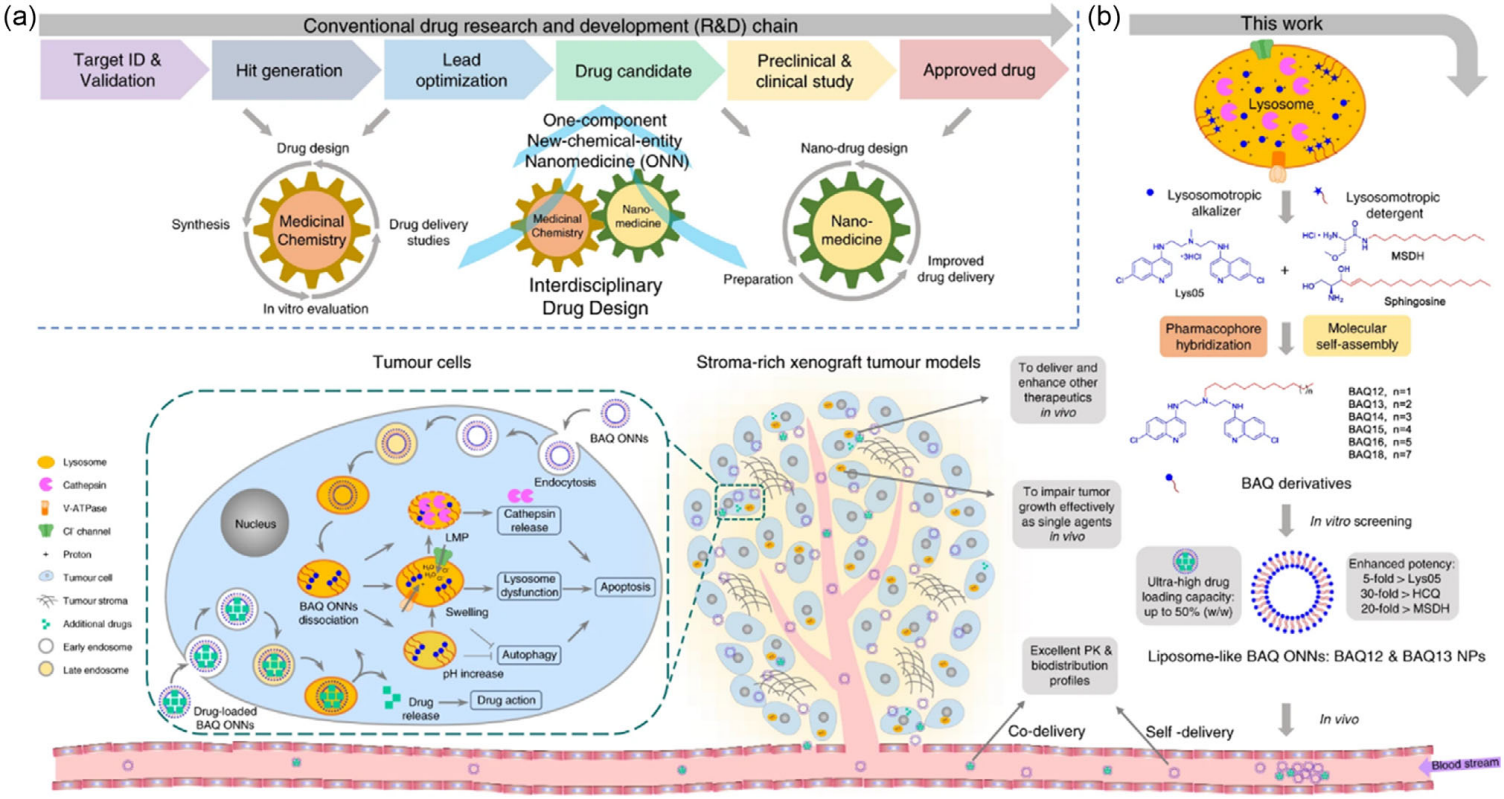
a) An interdisciplinary approach combining medicinal chemistry and nanomedicine is proposed to design ONNs. b) Self‐delivering lysosomotropic BAQ derivatives are created by hybridizing lysosomotropic detergents with BAQ‐based autophagy inhibitors to form self‐assembling BAQ ONNs. These ONNs exhibit enhanced in vitro activity, excellent delivery profiles, and significant in vivo therapeutic effects as single agents. They also offer high drug‐loading efficiency for additional therapeutic agents, making them a promising tool for combination therapy. Reproduced under the terms of the CC BY license.^[^ ^110^ ^]^ Copyright 2020, The Authors. Published by Nature Portfolio.

Li team introduced a novel self‐delivering one‐component new‐chemical‐entity nanomedicine (ONN) strategy for cancer therapy.^[^ ^110^ ^]^ These ONNs were created by hybridizing a lysosomotropic detergent (MSDH) with the autophagy inhibitor Lys05 to form bisaminoquinoline derivatives (BAQs) (**Figure** **5**).^[^ ^110^ ^]^ BAQ ONN can self‐assemble into NPs alone or with aiding lipids and exhibit superior lysosomal disruption and autophagy inhibition compared to traditional agents such as HCQ and lys05, showing a 30‐fold and 5‐fold increase in antiproliferative activity, respectively. Additionally, BAQ ONNs can efficiently encapsulate other therapeutic agents (>85%) such as bortezomib, rapamycin, etoposide, apoptozole, vinblastine, napabucasin, lenalidomide, and DiD dye, which makes them promising candidates for combination therapies targeting autophagy in cancer treatment. Notably, the 100% ONN active pharmaceutical ingredient content supports straightforward synthesis and scalability, highlighting their potential for clinical translation. These nanotherapeutics demonstrated excellent pharmacokinetics, favorable safety profiles, and significant antitumor efficacy as single agents in vivo in mouse models of pancreatic (MIA PaCa‐2) and colon (HT29) tumors. Furthermore, ONN exhibited potent antitumor activity in combination with the STAT3 inhibitor napabucasin in mice‐bearing patient‐derived pancreatic cancer stem cells. This work emphasizes the significant role of BAQ ONNs as potent autophagy modulators that can enhance therapeutic outcomes in cancer therapy.

### 3.2 Nanomedicine as Carriers for Autophagy Modulation

Several platforms have been used to deliver autophagy modulators as single agents or in combination with other cancer therapies. These nanoplatforms, while not exclusive to autophagy modulators, provide versatile systems for their delivery, particularly in the context of different combination strategies.

NPs for drug delivery can be broadly categorized based on their composition and structural properties into lipid‐based NPs (e.g., liposomes, LNP, micelles, nanoemulsions), polymer‐based NPs (e.g., polymeric NPs (PLGA, chitosan, PEG, HA), polymeric micelles, dendrimers, and hydrogels), carbon‐based and inorganic NPs (metal and metal oxide NP, silica, QD) (see Section 3.1), protein‐based (Albumin, Ferritin), or hybrid, which combine two or more of the above categories.

Autophagy modulators, and other drugs, for that matter, can be incorporated into NPs following two broad strategies: coloading and postloading.^[^ ^270^, ^271^, ^272^ ^]^ Co‐loading is used for loading drugs into NPs during their formation. Various systems are accessible to most research labs and are suitable for most drugs, such as lipid‐based and polymer‐based NPs. These systems are suitable for hydrophilic and hydrophobic drugs and usually involve physical entrapment, hydrophobic, electrostatic, π–π interactions, and chemical conjugation.^[^ ^272^ ^]^ Drugs could be entrapped in the NP core or polymer matrix, adsorbed to the surface, or conjugated to other NP components (i.e., polymers, lipids, proteins, and other drugs).^[^ ^273^ ^]^ Lipid‐based NPs are typically compounded via physical methods relying on the self‐assembly properties of lipid molecules and surfactants or the application of external energy. Similarly, polymeric NPs are formed by self‐assembly but often involve varied degrees of chemical manipulation, such as crosslinking, conjugation, and polymerization, to form stable NPs with desired properties. Postloading strategies consist of incorporating drugs into preformed nanocarriers. This strategy is particularly useful for porous nanocarriers with large surfaces like carbon‐based or inorganic NPs. Many of these NPs are endowed with autophagy‐modulating properties but are often used as carriers to load other drugs and autophagy modulators. Regardless of the loading strategy, targeting ligands and stimuli‐responsive cleavable linkers could be introduced, and NPs can be coated with additional materials (polymers, lipids, biological membranes, etc.) to introduce novel functionalities or enhance properties such as stability, biocompatibility, blood circulation time, targeting, and controlled release.

**Table** **3** and **4** present recent NPs targeting autophagy modulation, both inhibition and promotion, along with the rationale for their combination strategies.

**Table 3:** Combinations using nanomaterials for autophagy inhibition in cancer therapy.

Nanomateriala)	Autophagy modulator	Combination (strategy)	Cancer	Model	Rationale/Effect	References
PLGA coated with GR9 and DMA‐modified DSPE‐PEG.	CQ GR9 (autophagy‐responsive CPP)	DTX (Chemo)	Melanoma	B16F10/C57 mice	DMA‐DSPE‐PEG is hydrolyzed in the tumor's acidic pH, exposing cationic GR9 for better solid tumor penetration. Strong autophagy then cuts off GR9. CQ is released and synergizes with DTX to enhance anti‐tumor activity.	[292]
PLGA coated with TRAIL membrane vesicles (TH‐NP)	HCQ (inhibition)	OXA (Chemo)	HCC	HCCLM3, HepG2, and Huh‐7 metastatic mouse models.	TH‐NP efficiently suppresses tumor growth and tumor metastasis. TH‐NPs actively accumulate in the tumor site through TRAIL binding to DR4/5 on HCC cells. Autophagic flux inhibition by HCQ sensitizes HCC cells to OXA.	[275]
MMP2‐responsive triblock polymer‐peptide NPs	HCQ	CPG (Immuno)	Melanoma Colorectal (TLR9+)	B16, CT26/(SC) C57/BL6	CpG‐loaded NPs induce TLR9‐mediated ACD and elicit immunogenicity in TLR9‐positive tumors. They effectively reprogram the tumor's immunosuppressive microenvironment and suppress tumor growth and recurrence.	[288]
Polymeric NP (P‐PD‐L1‐CP)	PDPA polymer	Anti‐PD‐L1 (Immuno)	Prostate	TRAMP‐C2, (SC) C57BL/6 m	The PDPA core blocks autophagy and increases MHC‐I expression, making cancer cells vulnerable to TNF‐α and CTLs. P‐PD‐L1‐CP enhances tumor accumulation, promotes immune cell maturation, and inhibits tumor growth.	[462]
Polymer complex (PEI‐ oleic acid)	CQ	Atezolizumab (Anti‐PD‐L1) OVA, CPG (Immuno) PTX (Chemo)	Breast	4T1/(SC) BALB/c mice	Atezolizumab neutralized PD‐L1, activating immune responses, while chondroitin sulfate improved cell uptake and targeting. The platform delivered PTX and CQ to tumors, OVA, and CpG lymph nodes. CQ reversed the immunosuppressive TME by inhibiting autophagy.	[297]
Alkaline‐LDH NPs	LDH NPs	–	Melanoma Colorectal	B16F10, CT26, (SC) C57BL/6 mice	LDH NPs demonstrated long‐lasting, effective acid neutralization in the TIME, obstructing lysosome‐mediated autophagy pathway and enhancing the presence of antitumor‐associated macrophages/T cells	[295]
Pt(IV)/CQ/PFH‐^D^PPA‐1 NP	CQ	PFH (US contrast agent) Pt(IV) prodrug (chemo) ^D^PPA‐1 (anti‐PD‐L1) (Immuno)	Breast	4T1/(O) BALB/C mice	pH‐ and GSH‐sensitive NPs US contrast agent (UCA) targeting PD‐L1 (^D^PPA‐1) and release payload at the tumor site. CQ inhibits the protective autophagy of Pt(IV). NPs boost mDC and M1 macrophage ratios in the TME. PFH enables US monitoring.	[459]
GSH‐responsive self‐assembled NP	HCQ	SN38 polymeric prodrugs (Chemo)	TNBC	4T1, MDA‐MB‐231, MDA‐MB‐453, BT549/(O)Balb/c mice	The combo NP (HCQ: SN38) had significantly better therapeutic outcome compared to both free drug combinations/single drug NPs	[463]
Liposomes	HCQ	PTX (Chemo) TH‐cRGD (pH‐sensitive ligand)	Pancreatic	BxPC‐3, NIH 3 T3/(O/SC) nude mice	High targeting and penetration in pancreatic tumors; effective tumor cell killing and reduction of stromal fibrosis, thereby enhancing the therapeutic efficacy.	[464]
Liposomes	TMP	DOX (Chemo) JY4 (PD‐L1 binding peptide)	Lung	LLC/(SC), C57BL/6 J	TMP‐loaded and PD‐L1‐targeting liposomes accumulate in the tumor and inhibit the autophagic flux (by TMP) to enhance the recruitment of immune cells in the tumor.	[465]
Liposomes	LY294002	5‐FU (Chemo)	Esophageal	EC 9706	Autophagy inhibition by LY294002 improved the sensitivity of cancer cells to 5‐FU.	[458]
Micelles	Wortmannin	Dox (Chemo)	Breast melanoma	4T1 B16F10	Size‐adjustable micelles showed efficient penetration and retention in tumors and significant anticancer effects. Wortmannin‐mediated autophagy inhibition enhanced Dox‐mediated cytotoxicity.	[466]
Micelles (CaP‐coated)	CQ	PD‐173074 (FGFR Inhibitor), AZD9291 (EGFR‐inhibitor) cRGD (targeting ligand)	Lung cancer	H1975/AR/(SC) Balb/c nude mice	CQ counteracted the protective autophagy triggered by both AZD9291 and PD‐173074. NPs achieved effective tumor targeting (EPR + cRGD), minimal toxicity, and excellent antitumor efficacy in mice.	[467]
Micelles (pH‐responsive hyperbranched polyacylhydrazon)	LY294002	DOX (Chemo)	Oral	HN‐6, CAL‐27	Selective release of LY294002 (autophagy inhibitor) suppressed autophagy in the tumor cells, increasing their sensitivity to DOX.	[284]
Micelles (PEG‐b‐PLGA)	CQ	DTX (Chemo)	Breast	MCF‐7/(SC) in SCID	CQ addition improved the cell‐killing effect of DTX 12‐fold	[279]
Lipid‐PLGA‐HA NPs	CQ	mRIP3‐pDNA	Colorectal	4T1B16/F10, CT26/(SC) Balb/c mice	CQ and RPI3 overexpression induced lysosomal membrane permeabilization (LMP) and cell death and suppressed tumor growth.	[468]
Chitosan‐TPP NPs	shATG‐5	Gefitinib (EGFR inhibitor)	Lung	A549, PLC/(SC) Balb/c nude mice	Autophagy inhibition by shATG‐5 enhanced Gefitinib, anti‐tumor efficacy	[364]
Chitosan‐PLA NPs	siP62 (P62 knockdown) pβ5 (expressed)	Cisplatin (EGFR inhibitor)	Ovarian	2008/C13	22% higher killing compared with Cisplatin alone.	[469]
Hydroxyethyl Starch NPs	HCQ	–	Pancreatic	MiaPaca‐1, MiaPaca‐2, AsPC‐1	NPs reduced migration and invasion more efficiently than equivalent doses of HCQ.	[470]
Carbon monoxide nano complexes (HMPOC@M)	MnCO and CBD (PDT)	–	Breast	4T1, (O), Balb/c mice.	CO donors release CO and Mn^2+^ in the TME, inducing ROS‐mediated apoptosis. HMPOC@M enhances autophagy, promoting cancer cell death and inhibiting autolysosome degradation. In vivo, HMPOC@M with laser inhibits tumor growth and metastasis by downregulating VEGF and MMP9.	[471]
PEG‐AuNPs	Gold (Au)	–	Liver	Hepa1‐6, RAW 264.7 (SC) BALB/c	PEG‐AuNPs suppressed TAM M2 polarization in vitro and in vivo by inhibiting autophagic flux.	[164]
HA‐coated ZIF‐8	CQ	Polyoxometalate (PTT)	Ovarian	SKOV3	PTT/anti‐inflammation/anti‐autophagy combinations for efficient ovarian cancer treatment	[472]
PEG‐Ag_2_S QDs	PEG‐Ag_2_S QDs	5‐FU (Chemo) Cetuximab (EGFR inhibitor)	Lung (EGFR‐positive)	A549	Selective delivery of 5FU to A549 cells enhanced cell death associated with apoptosis. PEG‐Ag_2_S suppressed the protective autophagy induced by 5FU.	[239]
SPN_CN_ (Semiconducting polymer complex)	CQ	NLG919 (Immuno) SPN_CN_ (PDT and ICD).	Melanoma	B16F10/(SC)C57BL/6	SPN_CN_, activated by NIR, generates ^1^O_2_ for PDT and ICD, releasing CQ and NLG919 (IDO1 inhibitor) in the tumor. CQ enhances PDT and ICD, while NLG919 targets immunosuppressive tryptophan metabolism, boosting antitumor immunity and inhibiting tumor growth.	[473]
LTANP, PNC coated with M6PL and VI and PEG	LTANP	*α*PD‐L1 (Immuno)	Melanoma	B16F10‐Luc (SC) C57BL/6	LTANPs cause LMP and DAMPs release leading to immune activation by promoting dendritic cell maturation, increasing CD8 + T‐cell infiltration, and reversing tumor immunosuppression. The combination of LTANP and *α*PD‐L1 to prevent tumor recurrence and metastasis	[109]
PAA/CaP NPs	–	Epirubicin (Chemo)	hepatocellular carcinoma (HCC)	VX2 tumors (O), rabbit	PAA/CaP NPs block autophagy through the increase of intracellular Ca2+ content, which synergistically enhances the toxicity of EPI. leading to enhanced Transarterial chemoembolization (TACE).	[474]

a) CaP, calcium phosphate; Chemo, chemotherapy; CPP, cell‐penetrating peptide; CQ, chloroquine; DAMPs, damage‐associated molecular patterns; DOX, doxorubicin, DTX, Docetaxel; EPR, enhanced permeability and retention; FGFR, fibroblast growth factor receptor; HA, hyaluronic acid; HCC, hepatocellular carcinoma; HCQ, hydroxychloroquine; LMP, lysosomal membrane permeabilization; Immuno, immunotherapy; M6PL, mannose‐6‐phosphate ligand; NIR, near‐infrared; O, orthotopic; PAA, poly(acrylic acid); PEI, polyethyleneimine; PDPA, poly (2‐diisopropyl aminoethyl methacrylate); PDT, photodynamic therapy; PLGA, poly (lactic acid*‐co*‐glycolic acid); QD, quantum dots; SC, subcutaneous; SCID, severe combined immunodeficient; TIME, Tumor Immune Microenvironment; TNBC, triple‐negative breast cancer; TPP, tripolyphosphate;VI, 1‐vinyl imidazole.

**Table 4:** Combinations using nanomaterials for autophagy induction in cancer therapy.

Nanomateriala)	Autophagy modulator	Combination (strategy)	Cancer	Model	Rationale/Effect	References
HA‐EPI and Arginine and PCL–PEG (STF@AHPPE)	STF	Epirubicin (Chemo) Arginine (CPP)	Colorectal	CT26/(SC) BALB/c mice	STF@AHPPE NPs induced cytotoxic autophagy and strong ICD efficacy. NPs accumulate in tumor tissues, enter cells via HA and Arg, and release EPI and STF upon disulfide bond cleavage in high GSH concentration. NP combination demonstrated superior in vivo efficacy compared to separate drug administration.	[98]
ZIF‐8 NPs (ZIF‐8)	STF	BMS202 (PD‐1/PD‐L1 inhibitor)	Liver	SMMC7721, Huh7, H22/(SC), BALB/c mice	Enhanced ICD in residual tumor cells, effective activation of the anti‐tumor immune microenvironment, and inhibited the growth of remaining tumors.	[298]
LNO nanozyme (Phosphatase‐like lanthanum nickel oxide)	NPs	–	Melanoma	B16F10, C57BL/6	LNO‐induced macrophage autophagy promotes M2‐to‐M1 polarization, reduces TAMs, and enhances antitumor immunity. Myeloid cell membrane coating further boosted efficacy.	[475]
Chiral polymer‐modified AuNPs (PAV‐AuNPs)	^D−^PAV‐AuNPs	–	Breast	MDA‐MB‐231, BALB/c nude mice	^D−^PAV‐AuNPs induce autophagy in a chirality‐dependent manner in MDA‐MB‐231 cells but suppress autophagy in normal cells like 3T3 fibroblasts and HBL‐100. This selective activation was due to differences in ROS generation, cellular uptake, and sustained autophagy stimulation.	[476]
Mesoporous magnetite (mFe_3_O_4_) and HA	Rapamycin	Dox (Chemo)	Breast	4T1/(IV) BALB/c	Rapamycin promotes autophagic death. The combination improved the anti‐tumor effect and inhibited lung metastasis.	[477]
PLGA‐PEG NPs	PTEN mRNA	–	Melanoma Prostate	B16F10, PTEN‐CaP8/(SC, O) C57BL/6	When combined with immune checkpoint inhibitors, NPs activated immune responses and reversed the immunosuppressive TME, enhancing antitumor effects and immunological memory.	[478]
CNPs	shATG‐5	Gefitinib (EGFR inhibitor)	Lung Liver	A549, PLC/(SC)BALB/c nude mice	shATG‐5 inhibited autophagy and caused higher cell killing and apoptosis when combined with gefitinib.	[364]
Liposomes	Dihydroartemisinin (DHA)	Epirubicin (EPI) (Chemo)	Breast	MDA‐MB‐435S, MCF‐7/(SC) BALB/c nude mice	DHA‐EPI liposomes induced excessive autophagy in breast cancer cells by reducing Bcl‐2, enhancing Beclin‐1 release, and activating Bax.	[274]
Mannosylated liposomes (Man‐lip)	Dihydroartemisinin (DHA)	Dox (Chemo)	Colorectal	HCT8/ADR (SC)/BALB/c nude mice	Man‐lip effectively inhibited drug‐resistant colon cancer growth, enhanced apoptosis, induced autophagy, and reversed MDR through targeted delivery to mannose receptor‐overexpressing cells.	[289]
Folate‐modified liposomes in poloxamer‐based hydrogel	Rapamycin	–	Bladder	MBT2/(O) C3H mice	Bladder intravesical delivery significantly inhibited tumor growth compared to rapamycin‐loaded conventional liposomes.	[479]
Platelet membrane‐coated black phosphorus QDs	Hederagenin (HED)	–	Breast cancer	MCF‐7, BALB/c	Improved HED delivery into breast tumors and enhanced its anti‐tumor efficacy by promoting mitochondria‐mediated cell apoptosis and autophagy via upregulation of Beclin‐1 and LC3‐II.	[240]
Lantanide (III) complexes	HQNO‐1,10‐phen	–	Lung	A549/DDP, mouse	Complex Ln1 increased LC3 and Beclin‐1, decreased p62, and triggered apoptosis in A549/DDP cisplatin‐resistant cells.	[480]
Grafted HA polymer (@PCL–PEG)	STF	Epirubicin (EPI), arginine (CPP)	Colorectal	CT26/(SC) BALB/c mice	NPs target tumor tissues, enter cells via HA and Arg, and release EPI and STF (autophagy inducer) upon disulfide bond cleavage in high GSH concentrations. They induced cytotoxic autophagy and strong ICD	[98]
Micelles coated with HA‐OXA	STF	OXA (Chemo) C‐TFG (autophagy‐responsive motif)	Colorectal	CT26/(SC) Balb/c mice	NPs trigger ICD and mild autophagy upon OXA release, activating ATG4‐mediated TFG cleavage and STF release. This enhanced autophagy, induced cell death, and boosted antigen processing, leading to strong immune stimulation and antitumor efficacy in mice.	[108]

a) Chemo, chemotherapy; CPP, cell‐penetrating peptide; DBPA, 3‐(dibutylamino)‐1‐propylamine; C‐TFG, cholesterol‐TFG peptide; D‐PPA, disulfide‐based polymeric prodrug assembly; HA, hyaluronic acid; HCC, hepatocellular carcinoma; HDDA, 1,6‐hexanediol diacrylate, hydrophobic monomer; HQNO‐1,10‐phen, 8‐hydroxyquinoline‐N‐oxide1,10‐phenanthroline; ICD, immunogenic cell death; Immuno, immunotherapy; IV, intravenous; MOF, metal‐organic framework; NPs, nanoparticles; PEG, polyethylene glycol; PEG‐PCL, polyethylene glycol–polycaprolactone; PLGA, poly(lactic*‐co*‐glycolic acid); STF, STF62247.

## 4 Nanomedicine in Autophagy‐Targeted Combination Therapies

Single‐agent cancer treatments often show limited effectiveness due to tumor heterogeneity, drug resistance, and toxicity. While single therapies may produce good initial responses, tumors frequently adapt over time, reducing long‐term efficacy. Additionally, single‐agent therapies often lack the synergy needed to target multiple pathways dysregulated in cancers, making combination therapies more effective for long‐term treatment. As discussed above, targeting autophagy in combination with conventional therapies can sensitize tumor cells to treatment, reduce the chances of relapse, and improve overall therapeutic outcomes. NP‐based delivery systems are particularly suited for such combination approaches, offering versatile platforms to codeliver diverse therapeutics (e.g., small molecules, proteins, and genetic materials) to modulate autophagy‐related pathways.^[^ ^267^, ^274^ ^]^

This section explores combination strategies using autophagy regulators to enhance cancer therapy, focusing on how autophagy modulation can improve the efficacy of chemotherapy, immunotherapy, phototherapy, gene therapy, and others.

### 4.1 Chemotherapy

Despite the emergence of novel approaches such as immunotherapy and cell therapy, chemotherapy remains a key component in many standard treatment regimens, along with surgery and radiation therapy. Nanomedicines help address key chemotherapy‐related challenges, such as poor drug bioavailability, inadequate tumor accumulation, drug resistance, and off‐target toxicity. They can overcome drug resistance through combination therapies, including autophagy modulators. Autophagy inhibitors like CQ and SAR405 sensitize cancer cells to chemotherapeutic agents by blocking autophagy as a survival mechanism. Alternatively, autophagy‐inducing NPs can enhance chemotherapy by triggering AMCD. Various NPs including lipid‐based, micelles, albumin‐based, carbon‐based, inorganic, and polymer‐based (e.g., chitosan, HA, collagen, gelatin, PLGA, cyclodextrin) have been utilized in autophagy‐targeting approaches.^[^ ^98^, ^275^, ^276^, ^277^, ^278^ ^]^

Coadministration of CQ and DTX‐loaded PEG‐b‐PLGA micelles (40 nm) resulted in a 12‐fold increase in cell killing and a strong tumor suppression in MCF‐7 subcutaneous breast cancer models, compared to the group without autophagy inhibition. This effect was attributed to cancer cell sensitization by countering autophagy induction caused by micelles.^[^ ^279^ ^]^ To address challenges of premature degradation and limited clinical use of the PIK3C3/Vps34 inhibitor SAR405, Alamassi et al. demonstrated that chitosan NPs (CNPs) loaded with SAR405 or CQ effectively inhibit autophagy by blocking autophagosome‐lysosome fusion. In A549 lung cancer cells, SAR405‐loaded CNPs significantly inhibited autophagy, enhancing the chemotherapy efficacy of coloaded DOX.^[^ ^280^ ^]^ pH‐driven NPs are designed to release therapeutic payload in response to pH change, taking advantage of the acidic environment in TME or within specific cellular compartments (i.e., lysosomes).^[^ ^281^ ^]^ This strategy can enhance treatment precision and reduce damage to surrounding healthy tissues.^[^ ^282^ ^]^ Wang et al. constructed pH‐responsive drug‐induced self‐assembled nanovesicles to co‐deliver DOX and CQ to Dox‐resistant human MCF7/ADR breast cancer.^[^ ^283^ ^]^ These nanovesicles increased intracellular drug concentration and inhibited P‐gp efflux, reversing multidrug resistance and promoting antitumor effects, likely due to downregulating autophagy by CQ. Saiyin et al. conjugated pH‐responsive hyperbranched polyacylhydrazone (HPAH) to hydrophobic DOX. The resulting amphiphilic HPAH‐DOX self‐assembled into micelles in an aqueous solution allowing the coloading of autophagy inhibitor LY204002. In response to the intracellular acidic environment, these NPs released LY204002 and DOX in oral cancer cells (HN‐6 and CAL‐27), enhanced cell apoptosis, and downregulated autophagy, improving DOX efficacy.^[^ ^284^ ^]^ Chen et al. developed 6‐phosphonohexanoic acid‐modified dendrigraft poly‐L‐lysine NPs (PDGL‐GEM) to load gemcitabine, a first‐line chemotherapeutic drug against PDAC. PDGL‐GEM NPs were coprecipitated with the autophagy inhibitor CQ phosphate and calcium phosphate to form PDGL‐GEM@CAP/CQ.^[^ ^285^ ^]^ Calcium phosphate increased NP uptake nearly twofold at pH 6.5 versus pH 7.4 and decreased cell toxicity to NIH3T3 fibroblast. The stronger anticancer activity of PDGL‐GEM@CAP/CQ compared with PDGL‐GEM@CAP in Pan 02 cells was attributed to CQ‐mediated inhibition of autophagosome‐lysosome fusion. In vivo, PDGL‐GEM@CAP/CQ significantly suppressed tumor growth in pancreatic xenografts and orthotopic models compared to formulations without GEM or CQ.

Lin et al. used a pH‐sensitive mPEG‐poly(amino ester) graft copolymer to encapsulate gold (Au I) and form micelle‐like NPs.^[^ ^286^ ^]^ These NPs accumulated in acidic lysosomes and disintegrated upon the protonation of tertiary amines in poly(β‐amino ester), leading to lysosome damage and further impairment of the autophagosome and lysosome fusion. Furthermore, Au (I) inhibits thioredoxin reductase and causes oxidative damage via ROS generation, leading to autophagy and apoptosis induction in breast tumors. It is worth noting that the effect of these polymeric NPs on autophagy is concentration dependent. Lin et al. reported that low concentrations of polymeric NPs promote autophagy by modulating mTOR signaling, while high concentrations can induce ACD.^[^ ^287^ ^]^ Wang et al. engineered stimuli‐responsive CpG‐loaded nanorobots that induce autophagy‐mediated therapy for toll‐like receptor 9 (TLR9) positive cancer therapy.^[^ ^288^ ^]^ These nanorobots were created by coself‐assembling two amphiphilic triblock polymer peptides: one with an MMP2‐cleavable GPLGVRGS motif for tumor targeting and controlled release and the other with an arginine‐rich GRRRDRGRS sequence to condense CpG payloads. This design enables effective delivery of CpG to TLR9‐positive tumors, inducing AMCD and boosting antitumor immunity.^[^ ^288^ ^]^

**Figure 6:**
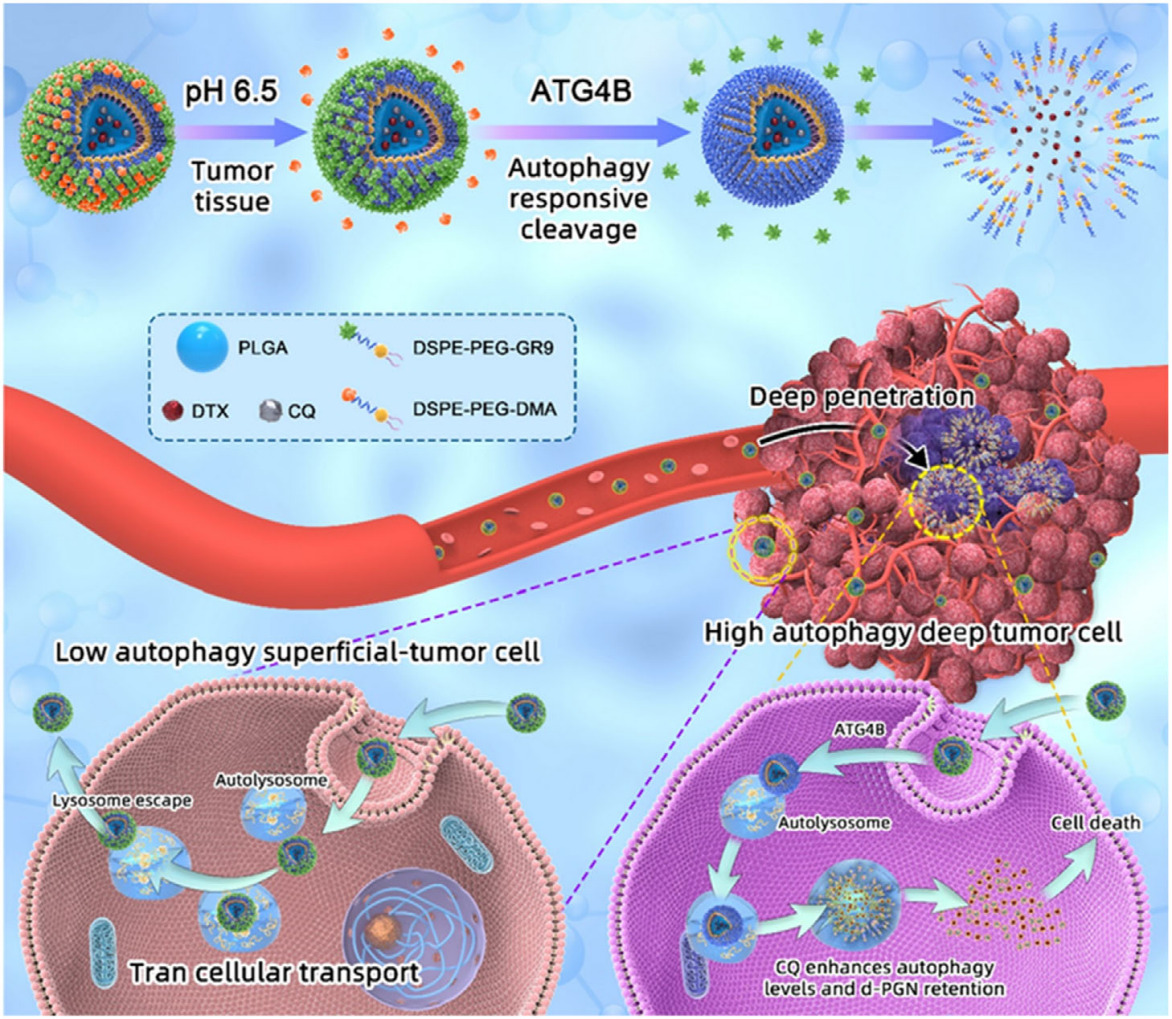
Schematic illustration of the self‐assembly and transportation pathway of autophagy‐responsive intraintercellular delivery NPs for effective deep solid tumor penetration. Reproduced under the terms of the CC BY license.^[^ ^292^ ^]^ Copyright 2022, The Authors. Published by BioMed Central.

NPs have also been utilized to deliver autophagy inducers, boosting the efficacy of cancer therapies. Colon cancer cells often resist DOX due to their aggressive behavior and high growth rate. Mannosylated liposomes containing DOX and dihydroartemisinin (DHA) were developed to overcome this.^[^ ^289^ ^]^ DHA is known to act in synergy with chemotherapeutics by inducing autophagy. Mannosylation enhanced NP selectivity for drug‐resistant HCT8/ADR cells overexpressing the mannose receptor, resulting in an 89% tumor inhibition rate. The combination reversed MDR through targeted delivery to mannose receptor‐overexpressing cells, improved DOX accumulation in the nucleus, increased apoptosis, and induced autophagy due to DHA. Dihydroartemisinin (DHA)‐loaded liposomes enhanced the circulation time of Epirubicin and improved its efficacy in different breast cancer cells by reducing Bcl‐2 activity, promoting Beclin‐1 release, and activating Bax. While Bax activation triggered apoptosis (type I death), DHA‐induced Beclin‐1 caused type II death via excessive autophagy. These findings suggest that by promoting autophagy, DHA could enhance epirubicin's anticancer activity.^[^ ^274^ ^]^ Black phosphorus QDs coated with platelet membranes were used to enhance the antitumor activity of Hederagenin (HED) to MCF‐7 breast tumor through autophagy stimulation via Beclin‐1 and LC3‐II.^[^ ^240^ ^]^ Polymeric NPs have recently been popular for their autophagy cascade amplification capability.^[^ ^98^, ^290^, ^291^ ^]^ Their surface properties can be optimized to enhance circulation time and improve tumor penetration and accumulation (**Figure** **6**).^[^ ^108^, ^292^ ^]^ Wang et al. developed an on‐demand autophagy cascade amplification NP (ASN) to boost oxaliplatin‐induced cancer immunotherapy.^[^ ^108^ ^]^ C‐TFG micelles are formed through the self‐assembly of amphiphilic peptide‐cholesterol monomers, where “C” refers to cholesterol and “TFG” is the peptide (GTFGFRRRRRRRR) containing a specific cleavage site “TFG” that could be recognized the autophagy enzyme ATG4, allowing for a controlled release of encapsulated payloads upon induction of autophagy. The core of C‐TFG micelles was loaded with the autophagy‐inducing drug STF‐62247, and the micelles were coated with HA prodrug (HA‐OXA) to form ASN. In the reducing TME, OXA is released, triggering tumor ICD and inducing mild autophagy. This activates the autophagy enzyme ATG4 which cleaves the C‐TFG micelle and releases STF‐62247, a potent autophagy inducer. ASN demonstrated optimal immune activation and enhanced antitumor efficacy in CT26 tumor‐bearing mice.

Recently, novel NPs have been designed as receptors to promote the selective autophagic degradation of oncoproteins. Xiaowan et al. developed a nanoreceptor (NRs) capable of binding to mutant p53 (mutp53) and directing it to the autophagosome to facilitate its degradation.^[^ ^107^ ^]^ NRs are composed of PEG–polylactic acid (PEG–PLA) NPs (≈100 nm) decorated with mutp53‐binding peptide (MBP) and cationic lipid (DOTAP). While MBP targets and binds to a broad spectrum of mutp53 but not the WT p53, the cationic lipid DOTAP increases autophagy levels by inducing increased autophagosome formation and directs NR‐bound mutp53 into autophagosomes, achieving efficient autophagic degradation of mutp53. The therapeutic potential of these novel NRs was demonstrated in ES‐2 tumor‐bearing mice and a PDX ovarian cancer model. NRs enhanced chemotherapy sensitivity, particularly when combined with cisplatin or other platinum‐based chemotherapy agents.

### 4.2 Immunotherapy

Autophagy plays a multifaceted role in cancer by influencing the tumor immune microenvironment (TIME) and regulating the immune system to either suppress or promote tumor progression.^[^ ^293^ ^]^ Autophagy could enhance tumor antigen‐presenting of multiple antigen‐presenting cells, such as dendritic cells and macrophages, and improve T cell recognition and activation against cancer cells. In addition, autophagy regulates cytokine release, immune cell recruitment, and macrophage polarization between pro‐tumorigenic M2 and anti‐tumorigenic M1 states within the TME. Autophagy regulation is essential for the effectiveness of recent immunotherapies, such as immune checkpoint inhibitors, which influence T cell function, the inflammatory response, and the mechanism of ICD. In contrast, it can also contribute to immunotherapy resistance by upregulating immune checkpoints such as PD‐L1, suppressing inflammatory cytokines (e.g., IFN‐γ), and promoting T‐cell exhaustion.^[^ ^294^ ^]^ Combining autophagy modulators with immune checkpoint inhibitors or immunostimulatory agents has shown promise in improving immune activation, overcoming resistance, and sustaining antitumor effects.

Given the critical role of autophagy in cancer immunity, several studies have explored the use of NPs to combine autophagy modulators with immunotherapy, aiming to enhance efficacy and overcome resistance. Zhang et al. developed weakly alkaline layered double hydroxide NPs (LDH NPs) to neutralize excess acid in the TME and block autophagy in tumor cells for neoadjuvant immunotherapy in melanoma and colon tumors.^[^ ^295^ ^]^ Peritumoral injection of LDH NPs induced an efficient long‐term acid neutralization in TIME, blocked lysosome‐mediated autophagy in tumor cells, and increased the levels of antitumor TAMs and T cells.^[^ ^295^ ^]^ Another approach is to shift TAMs from the protumor M2 phenotype to the antitumor M1 phenotype. Studies have shown that inorganic nanomaterials like AuNPs are more likely to be internalized by M2 macrophages.^[^ ^296^ ^]^ The immunomodulatory effect of PEG‐coated AuNPs was exploited to induce lysosome dysfunction and inhibit autophagic flux, suppressing M2 polarization in hepatoma Hepa1‐6 cells.^[^ ^164^ ^]^

Unlike traditional chemotherapy resistance, the mechanism of immunotherapy resistance is primarily due to the “loss or deficiency” of antigens. Therefore, several studies have focused on combining immunotherapy with other strategies to boost anticancer efficacy. Cheng et al. used a multilayered nanoplatform for targeted tumor and lymph node delivery in breast cancer therapy.^[^ ^297^ ^]^ A polymer complex of polyethyleneimine and oleic acid (PEI‐OA) was used to encapsulate PTX, CQ, ovalbumin antigen (OVA), and the CpG immunopotentiator. The resulting NPs were coated with atezolizumab (anti‐PD‐L1 antibody) and chondroitin sulfate. Atezolizumab neutralized PD‐L1 and activated immune responses, while chondroitin sulfate enhanced cell uptake and targeting of PD‐L1 and CD44. The platform efficiently delivered PTX and CQ to the tumor and OVA and CpG to the draining lymph nodes. Additionally, CQ helped reverse the immunosuppressive TME by inhibiting autophagy, which enhanced the effectiveness of both chemotherapeutics and immune checkpoint inhibitors.

**Figure 7:**
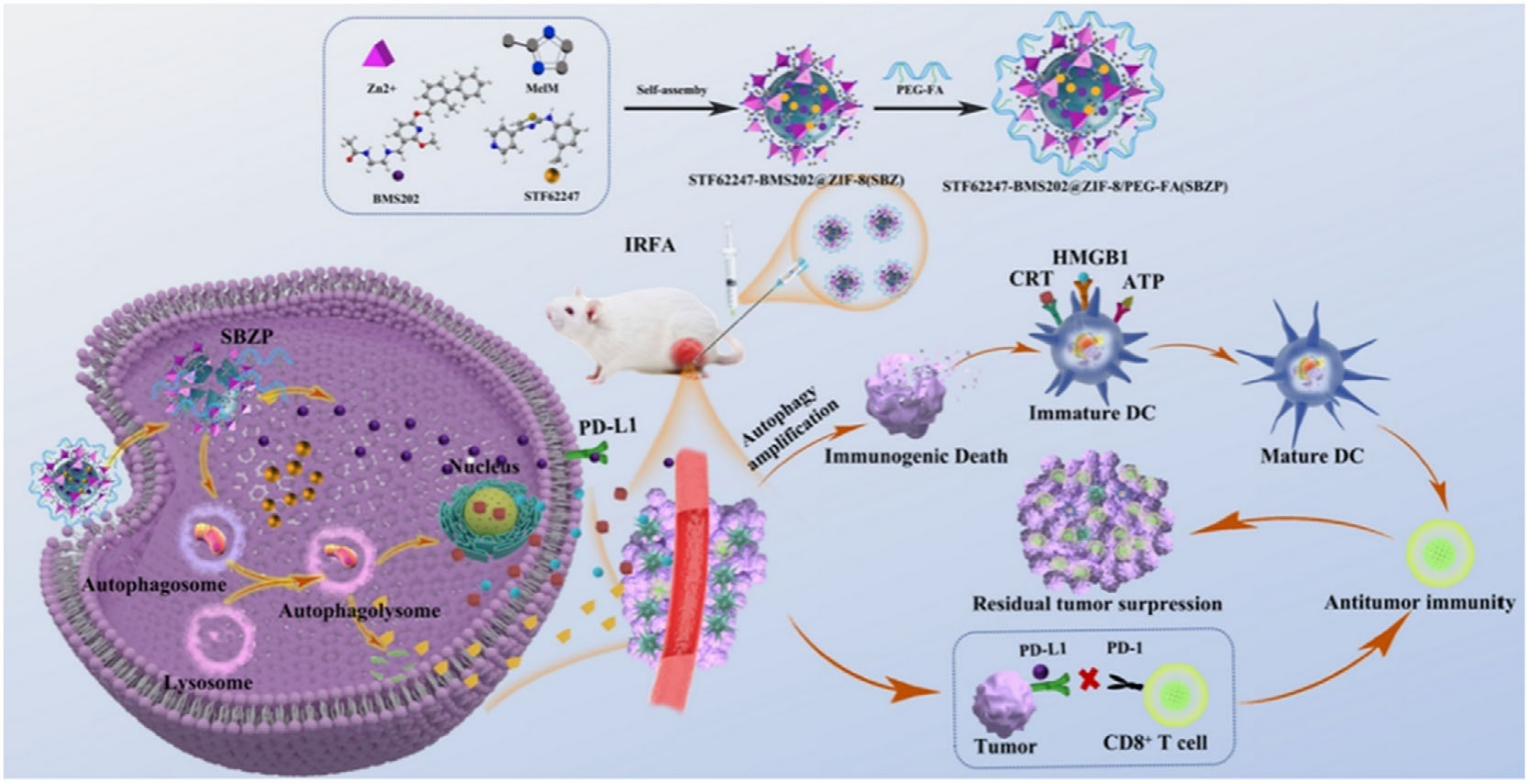
STF‐BMS@ZIF‐8/PEG‐FA induces ICD in synergy with anti‐PD‐1/PDL1 therapy to remodel the immune microenvironment following IRFA. Reproduced under the terms of the CC BY license.^[^ ^298^ ^]^ Copyright 2023, The Authors. Published by Springer Nature.

Autophagy inducers have been used to promote the ICD by exacerbating the mild protective autophagy provoked by incomplete radiofrequency ablation (IRFA). Zhang et al. developed ZIF‐8 NPs (SZP NPs) loaded with autophagy inducer STF62247 to overcome the immunosuppressive TME (**Figure** **7**).^[^ ^298^ ^]^ These NPs promoted autophagy‐dependent cell death by increasing autophagic flux, autophagosomes, and autolysosomes, inhibiting residual tumor growth in sublethally heated SMMC7721 and Huh7 cells. Furthermore, SZP NPs induced ICD in the IRFA subcutaneous H22 model by promoting dendritic cell maturation in residual tumors, enhancing CD4+ and CD8 + T cell infiltration, and increasing anti‐tumor cytokine secretion (IFN‐γ, TNF‐α) and effector memory T cells. Authors also constructed SBZP NPs by encapsulating autophagy inducer STF62247 and BMS202, a novel small molecule PD‐1/PD‐L1 inhibitor. Although SZP and SBZP initially performed well in inhibiting tumors, the SZP‐treated mice showed regrowth of residual tumors at 22 days post‐treatment. However, the SBZP group exhibited a sustained suppression of residual tumors in response to anti‐PD‐1/PD‐L1 therapy. These findings highlight the utility of combining immunotherapy with autophagy regulators.

Xing et al. designed LTANP to induce lysosomal membrane permeabilization (LMP) and trigger ICD.^[^ ^109^ ^]^ LTANP consists of a protein nanocapsule (PNC, albumin in this case) core with mannose‐6‐phosphate ligand (M6PL) and 1‐vinyl imidazole (VI) and is coated by a pH‐responsive PEG shell. The PEG shell detaches in the acidic TME, and LTANPs are taken up and accumulate in lysosomes where VI is protonated and crosslinked with negatively charged M6PL, causing PNC aggregation. This leads to LMP, impairing the autophagy‐lysosome pathway and releasing damage‐associated molecular patterns (DAMPs). DAMPs enhance immune activation by promoting dendritic cell maturation, increasing CD8 + T‐cell infiltration, and reversing tumor immunosuppression. Additionally, LTANP upregulates PD‐L1 expression, making tumors more susceptible to PD‐L1 checkpoint blockade. The combination of LTANP and *α*PD‐L1 enhanced antitumor immunity, induced long‐term immune memory, and prevented tumor recurrence and metastasis, offering a promising strategy for cancer treatment.

It is worth noting that ongoing clinical trials are assessing the combination of HCQ with immunotherapeutic agents, including Aldesleukin (IL‐2), nivolumab (anti‐PD‐1) alone and in conjunction with ipilimumab (anti‐CTLA‐4), and Avelumab (anti‐PD‐L1).

### 4.3 Photodynamic Therapy

Photodynamic therapy (PDT), a relatively noninvasive localized treatment,^[^ ^299^ ^]^ is now clinically applied in various cancers, including head and neck cancer,^[^ ^300^, ^301^ ^]^ bladder cancer,^[^ ^302^, ^303^ ^]^ glioblastoma,^[^ ^304^, ^305^ ^]^ and NSCLC.^[^ ^306^, ^307^ ^]^ The efficacy of PDT primarily depends on the generation of ROS by the photosensitizer upon light activation, which induces oxidative stress and ultimately leads to cell death.^[^ ^308^ ^]^ As a catabolic process in response to external and internal stress, autophagy can serve both as a survival mechanism and a pathway for promoting cell death in PDT.^[^ ^309^ ^]^ Nanotherapeutics combining autophagy modulation with PDT to treat cancers have been studied.^[^ ^310^, ^311^, ^312^ ^]^

**Figure 8:**
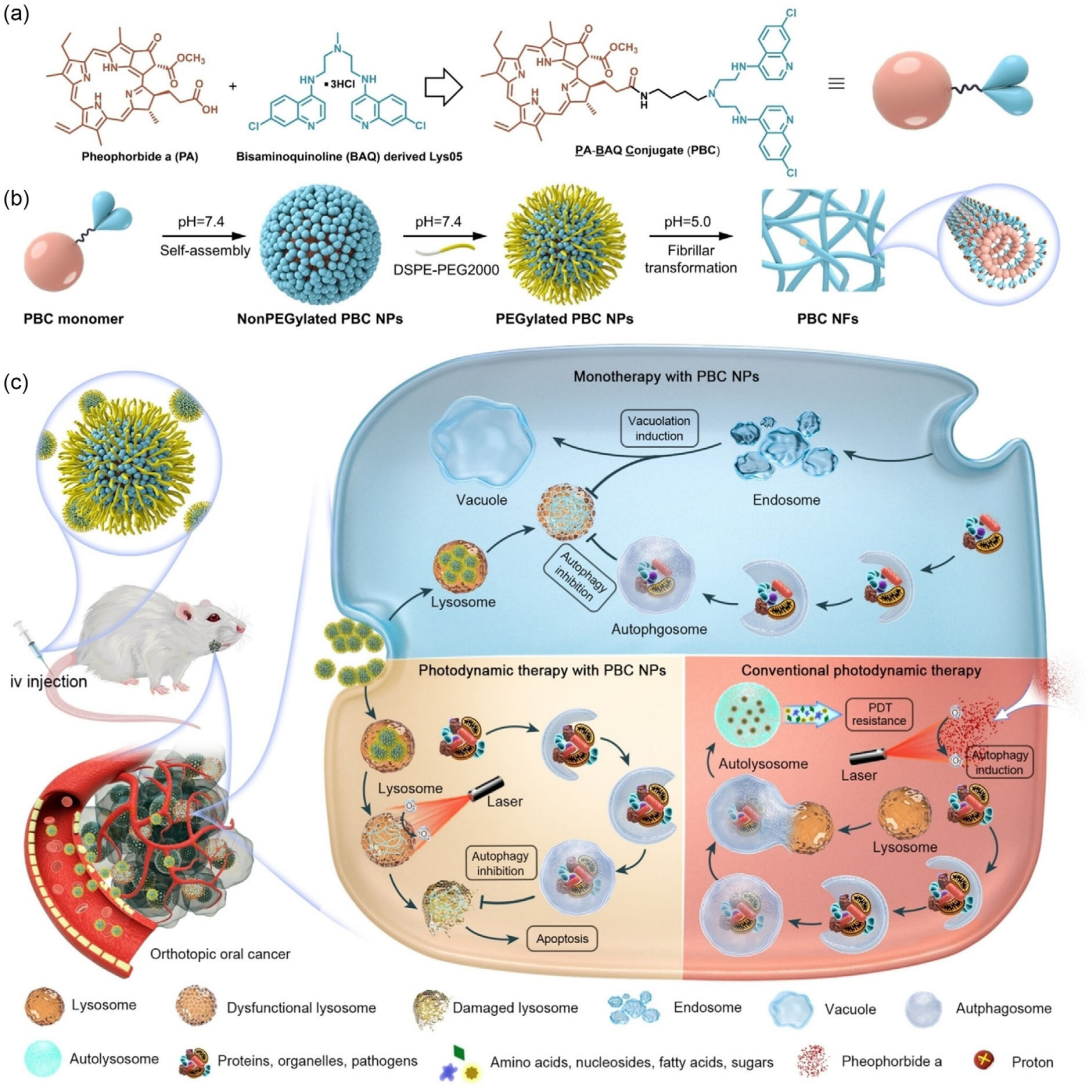
Schematic illustration of the lysosomal pH‐responsive small‐molecule‐based nanotransformer. a) Design of the PBC monomer. b) PBC self‐assembly and transformation at acidic pH (5.0). c) PBC NPs induce cancer cell death through lysosomal dysfunction, autophagy inhibition, and cytoplasmic vacuolization. PBC NPs also mediate PDT, overcoming autophagy‐related resistance in conventional PDT for effective tumor ablation. Reproduced with permission.^[^ ^110^ ^]^ Copyright 2022, Wiley.

During PDT, the generation of ROS often triggers autophagy as a cellular defense mechanism, enabling cancer cells to survive by clearing damaged organelles and proteins resulting from oxidative stress. This process may lead to resistance against PDT.^[^ ^313^, ^314^, ^315^, ^316^ ^]^ Therefore, inhibiting autophagy can block this protective mechanism and increase the vulnerability of cancer cells to PDT‐induced cell death. For instance, a recent study developed a pH‐driven small‐molecule nanotransformer (PBC) to address autophagy‐induced PDT resistance in cancer cells.^[^ ^111^ ^]^ PBC, a conjugate of pheophorbide A and BAQ, self‐assembles into NPs at physiological pH and transforms into nanofibrils within the acidic environment of lysosomes in tumor cells.^[^ ^110^ ^]^ This structural transformation disrupts lysosomal function and inhibits autophagy, hampering the ability of cancer cells to repair PDT‐induced damage (**Figure** **8**). The self‐assembling nanotransformer significantly increases the susceptibility of cancer cells to PDT, achieving a 100% cure rate in orthotopic oral cancer models after just two doses. This study highlights the potential of lysosome‐targeting strategies and autophagy inhibition in overcoming PDT resistance, ultimately enhancing the therapeutic efficacy of PDT in cancer treatment.

Conversely, excessive or dysregulated autophagy can lead to ACD, causing a shift in autophagy from prosurvival to prodeath.^[^ ^316^, ^317^, ^318^ ^]^ Consequently, autophagy inducers have also been shown to enhance PDT's effectiveness synergistically. 3‐bromopyruvate (3‐BP), an autophagy promoter and hypoxia ameliorator, has been incorporated into chlorin e6 (Ce6)‐encapsulated NPs to enhance the efficacy of PDT in treating hypoxic tumors.^[^ ^319^ ^]^ In addition, the autophagy promoter celastrol and photosensitizer Ce6 were found to self‐assemble into uniform NPs (CeCe) through *π*–*π* stacking and hydrophobic interactions. In vitro, results revealed that PDT‐induced autophagy maintained a basal cytoprotective level in CT26 cells. However, this carrier‐free nanotherapeutic induced excessive autophagy, reversing the cytoprotective response and promoting ACD. In vivo experiments further demonstrated that CeCe effectively overcame tumor resistance to PDT, significantly boosting synergistic tumor suppression.^[^ ^320^ ^]^ Additionally, some nanosensitizers possess intrinsic autophagy‐modulating properties. Upon light activation, nitrogen‐doped titanium dioxide NPs (N‐TiO2 NPs) have been shown to induce protective autophagy at low doses and switch to AMCD at higher doses in melanoma cells.^[^ ^321^ ^]^ These findings highlight the potential of nanosensitizers not only for their primary photodynamic effects but also for their ability to regulate autophagy, optimize PDT efficacy, and enhance therapeutic outcomes.

Although the role of autophagy in PDT is difficult to predict, as its impact depends on factors such as the type of cancer, light and drug dose, and the cellular environment, targeting autophagy holds promise for enhanced PDT.^[^ ^316^, ^322^ ^]^ Additionally, autophagy in PDT has been linked to modulating the immune response.^[^ ^323^ ^]^ Therefore, further investigation is needed to understand the interaction between autophagy and immune responses following PDT and how different tumor environments and cancer types respond to autophagy modulation during PDT, particularly in clinical settings.

### 4.4 Photothermal Therapy

PTT relies on photothermal agents converting near‐infrared (NIR) light into heat, inducing hyperthermia, which selectively kills cancer cells.^[^ ^1^ ^]^ Unlike PDT, which requires oxygen to produce ROS for cell death, PTT uses heat generated by absorbing light, making it effective even in oxygen‐deprived tumors. However, similar to PDT, autophagy is often activated in response to the heat stress caused by PTT, enabling cancer cells to remove damaged components and potentially leading to resistance to therapy.^[^ ^324^ ^]^ On the other hand, intense heat can also trigger excessive autophagy, resulting in ACD.^[^ ^325^ ^]^

A common strategy to enhance PTT efficacy is to coencapsulate autophagy inhibitors within nanotherapeutics. For example, the autophagy inhibitor CQ was incorporated into mesoporous polydopamine NPs (mPDA NPs) to create a biomimetic nanoplatform.^[^ ^326^ ^]^ The mPDA core offers effective photothermal capability, while NIR‐triggered release of CQ inhibits the PTT‐induced protective autophagy in prostate cancer cells, thereby reducing their resistance to PTT. This combined PTT and autophagy inhibition approach leads to significant autophagosome accumulation, ROS production, mitochondrial damage, ER stress, and apoptotic signaling, resulting in enhanced prostate tumor ablation in vivo.

In a related study, indocyanine green (ICG)‐mediated mild low‐temperature PTT (LTPTT) stimulated prosurvival autophagy in tumor cells.^[^ ^327^ ^]^ To counter this effect, CQ was introduced to overcome autophagy‐induced resistance in LTPTT. ICG and CQ self‐assemble into NPs via hydrophobic interactions, π–π stacking, and electrostatic interactions. NPs are then coated with a red blood cell–cancer hybrid membrane for prolonged circulation and tumor‐targeting capabilities. CQ inhibits protective autophagy during LTPTT by blocking the fusion of autophagosomes with lysosomes, thereby preventing the degradation of harmful metabolic products caused by photodamage and amplifying tumor cell damage during LTPTT.

PTT shows limited effectiveness against tumor cells that have colonized bone tissue. To address this, ZIF‐8 NPs encapsulating autophagy activator curcumin and liquid metal, functionalized with HA/alendronate (CLALN), were developed.^[^ ^328^ ^]^ CLALN exhibits pH‐responsive decomposition, releasing curcumin, which triggers mild autophagy. Upon mild heating, the combination of curcumin and PTT induces “overactivated autophagy,” leading to massive cell death and inhibiting the immunosuppressive microenvironment. This combination strategy of autophagy activation and mild PTT offers a promising approach to addressing the limitations of traditional PTT. In addition, a PDA‐based NP system was developed to enhance PTT in cancer cells by inducing autophagy.^[^ ^329^ ^]^ The PDA NPs were modified with PEG for stability and further functionalized with Beclin‐1‐derived peptides to stimulate autophagy and RGD peptides for enhanced tumor targeting. Upon NIR light irradiation, the PDA NPs generated hyperthermia to induce PTT. Meanwhile, the Beclin‐1‐derived peptides promoted autophagy in tumor cells, sensitizing them to heat‐induced damage. Beyond combining autophagy modulators with PTT, certain PTT materials such as gold‐based NPs^[^ ^157^, ^330^ ^]^ and iron oxide NPs^[^ ^331^ ^]^ possess inherent autophagy‐modulating properties and have been shown to regulate autophagy and enhance the effectiveness of PTT.

The combination of PTT and autophagy modulation has shown encouraging results in preclinical cancer models, but several areas require further investigation. One important aspect is the influence of thermal dose (temperature and duration) on autophagy pathways, as mild‐ and high‐temperature PTT may trigger different autophagic responses, affecting treatment outcomes. Additionally, while most studies on PTT are in the preclinical phase, more clinical trials are needed to assess the safety, efficacy, and potential side effects of autophagy modulation combined with PTT.

### 4.5 Sonodynamic Therapy

Sonodynamic therapy (SDT) is a noninvasive cancer treatment modality that uses ultrasound (US) to activate a chemical compound known as a sonosensitizer. This results in the generation of ROS to kill cancer cells.^[^ ^332^, ^333^ ^]^ Similar to PDT, SDT relies on ROS production but employs the US instead of light. Depending on the frequency and intensity, the US can reach depths of up to 10 cm or more compared to PDT's shallow penetration of less than 1 cm, making SDT a promising option for treating deeper‐seated tumors.^[^ ^334^, ^335^, ^336^, ^337^ ^]^

As SDT relies on ROS to induce cancer cell death, it faces the same challenge as other ROS‐based therapies, such as PDT, where ROS can trigger autophagy as a repair mechanism, leading to therapeutic resistance. Therefore, combining autophagy inhibition with SDT offers a potential strategy to enhance therapeutic efficacy.^[^ ^338^, ^339^ ^]^ A study has shown that sonosensitizer‐augmented SDT can induce cytoprotective autophagy by activating the MAPK signaling pathway and inhibiting the AMPK pathway.^[^ ^340^ ^]^ Liposomes coencapsulating the sonosensitizer protoporphyrin IX (PpIX) and the early‐phase autophagy‐blocking agent 3‐methyladenine (3‐MA) were developed. 3‐MA prevents autophagosome formation by regulating the phosphoinositide 3‐kinase (PI3K) pathway, significantly reducing cellular resistance to oxidative stress. This combination enhanced SDT‐mediated cancer cell apoptosis and demonstrated a remarkable synergistic effect, achieving 90% tumor inhibition in breast cancer models. CQ and HCQ‐loaded nanoplatforms were also reported to incorporate sonosensitizer to enhance SDT efficiency.^[^ ^341^, ^342^ ^]^

Since excessive or dysregulated autophagy can transition from a protective response to a cell death pathway, converting autophagy in this way presents a valuable approach to enhance the effectiveness of SDT. A notable example is the engineering of an oxygen economizer by conjugating the respiration inhibitor 3‐bromopyruvate (3‐BP) with hollow mesoporous organosilica NPs.^[^ ^341^ ^]^ These NPs were loaded with the sonosensitizer hematoporphyrin monomethyl ether and further modified with poly(ethylene glycol) (PEG) to improve their biocompatibility. 3‐BP inhibits cellular respiration, alleviating tumor hypoxia and inducing excessive autophagy. This overactivation of autophagy promotes cell death and significantly boosts SDT's therapeutic efficacy in vivo.

Tumor hypoxia is a well‐known challenge in both SDT and autophagy regulation. Engineering nanosonosensitizers to enhance oxygen delivery or reduce oxygen consumption at the tumor site could improve treatment outcomes. Furthermore, standardized protocols for SDT, including device optimization and US parameters (e.g., frequency and intensity), require further development. Sonosensitizers must also undergo rigorous testing to evaluate their pharmacokinetics, biodistribution, and biocompatibility to ensure safety and effectiveness in clinical settings.

### 4.6 Chemodynamic Therapy

Capitalizing on the elevated endogenous hydrogen peroxide (H_2_O_2_) levels in TME, CDT employs metal‐based agents to catalyze the conversion of H_2_O_2_ into cytotoxic hydroxyl radicals (•OH) through Fenton or Fenton‐like reactions, thereby inducing cancer cell death.^[^ ^343^, ^344^ ^]^

This approach offers tumor specificity and minimal side effects, as it does not require external stimuli. However, CDT‐induced oxidative stress often activates autophagy, allowing cancer cells to degrade damaged components and develop resistance to therapy.^[^ ^197^, ^344^ ^]^ To overcome this adaptive mechanism, codelivery of autophagy inhibitors within nanotherapeutics has emerged as a promising strategy to enhance CDT efficacy by disrupting the protective role of autophagy in cancer cell survival. Gu et al. developed metal–DNA nanocomplexes (DACs‐Mn) to enhance CDT by inhibiting autophagy‐mediated resistance.^[^ ^345^ ^]^ A cyclic DNA template containing complementary sequences for the AS1411 aptamer and ATG5 DNAzyme was utilized to construct DACs‐Mn. At the same time, Mn^2+^ ions were employed to assist in folding the long single‐stranded DNA into stable nanocomplexes. The AS1411 aptamer enabled specific recognition of tumor cells by binding to nucleolin receptors, promoting cellular uptake. Once internalized, the acidic intracellular environment degraded DACs‐Mn, releasing Mn^2+^ ions and DNAzymes. The released Mn^2+^ ions catalyzed the Fenton reaction, generating hydroxyl radicals (•OH) to induce oxidative damage, while the ATG5 DNAzyme specifically cleaved ATG5 mRNA, effectively inhibiting autophagy and preventing tumor cells from resisting CDT‐induced oxidative stress. Notably, DACs‐Mn demonstrated significantly enhanced efficacy compared to DACs‐Mg (without Mn^2+^) and DCs‐Mn (without ATG5 DNAzyme), exemplifying the synergistic effect of combining CDT with autophagy inhibition.

#### 4.6.1 Combination of PDT, PTT, and CDT

Individual treatments, such as PDT, PTT, and CDT, work through distinct mechanisms, each with inherent limitations that may allow cancer cells to develop alternative resistance pathways. For example, PDTs rely on oxygen to generate ROS, making them ineffective in hypoxic environments.^[^ ^346^, ^347^ ^]^ Therefore, integrating these therapies can induce various forms of cellular damage and simultaneously disrupt multiple survival pathways in cancer cells, thereby maximizing therapeutic efficacy. For instance, combining PTT and CDT leverages hyperthermia and ROS generation, enhancing cytotoxic effects. Moreover, since autophagy is frequently activated as a survival response to therapy‐induced stress, further inhibition can improve treatment outcomes by preventing cancer cells from escaping therapy‐induced damage. In a recent study, HCQ was encapsulated within hollow copper sulfide NPs (HCuS NPs) to form HCQ/HCuS (HCHP) NPs designed to enhance PTT and CDT by inhibiting autophagy‐mediated resistance.^[^ ^348^ ^]^ Once internalized by tumor cells, HCHP NPs interact with intracellular GSH, reducing Cu^2+^ to Cu^+^. The generated Cu^+^ then catalyzes a Fenton‐like reaction with overexpressed H_2_O_2_, producing highly reactive •OH that induces oxidative stress and cancer cell apoptosis. Additionally, the PTT effect of HCHP NPs further promotes the Fenton reaction, amplifying ROS generation and achieving a synergistic PTT–CDT therapeutic effect. Furthermore, HCQ is released from HCHP NPs upon laser irradiation, elevating lysosomal pH and disrupting lysosomal degradation. This inhibition of protective autophagy prevents cancer cells from mitigating oxidative and thermal stress, enhancing tumor cell sensitivity to PTT‐CDT therapy and improving overall therapeutic efficacy.

### 4.7 Gene Therapy

Regulating autophagy‐related genes has been considered for modulating autophagy in cancer. Approaches like CRISPR/Cas9, small interfering RNAs (siRNAs), microRNA (miRNA), and short hairpin RNAs (shRNAs) can target specific gene expression to suppress cancer progression.^[^ ^349^ ^]^ CRISPR/Cas9 technology uses single‐stranded RNA (sgRNA)‐guided Cas9 to precisely edit DNA, enabling the knockout of key autophagy genes and inhibiting autophagy protein expression.^[^ ^349^ ^]^ For example, ATG5 knockout has been shown to enhance atorvastatin‐induced cytotoxicity, inhibiting the progression of liver and colorectal cancer.^[^ ^350^ ^]^ However, efficient in vivo delivery of CRISPR/Cas9 for gene editing is challenging due to physiological barriers and the system's large size, complicating its safe delivery to the nucleus. RNA interference (RNAi) is widely used to modulate gene expression in most eukaryotic cells.^[^ ^351^ ^]^ shRNA and siRNA can target the transcripts of autophagy genes and have been used to inhibit the synthesis of autophagy proteins.^[^ ^352^, ^353^, ^354^ ^]^ For example, siRNA targeting ATG7 was combined with DTX for the treatment of breast cancer.^[^ ^355^ ^]^ ShRNAs have similar functions to siRNAs; shRNA can be processed into siRNA once inside the cell.^[^ ^356^ ^]^ In one example, shRNA of insulin‐like growth factor 1 receptor (IGF1R) was shown to upregulate LC3B expression, activating autophagy and inhibiting prostate cancer progression.^[^ ^357^ ^]^

RNA delivery faces several challenges, including RNA hydrophilicity and negative charge, large molecular size, instability, nuclease degradation, inefficient endosomal escape, immunogenicity, off‐target toxicity, and generally insufficient distribution in target tissues other than the liver.^[^ ^358^, ^359^, ^360^, ^361^, ^362^ ^]^ Most of these issues can be addressed through RNA modification and nanobased delivery systems, including lipid‐based NPs, amine‐functionalized or cationic polymers (e.g., PEI, PLL, chitosan), and lipid–polymer hybrids. Among these, LNPs are the leading, clinically proven nonviral nucleic acid delivery platform.^[^ ^363^ ^]^

Coloading gefitinib and shRNA‐expressing plasmid DNA targeting ATG‐5 gene (shATG‐5) into CNPs resulted in marked autophagy inhibition and suppressed tumor growth in mice bearing PLC tumors.^[^ ^364^ ^]^ Similarly, lipid‐polycation‐HA NPs loaded with metformin and VEGF siRNA demonstrated good antitumor effects by inhibiting the mTOR pathway and activating tumor autophagy in human NSCLC H460 tumor‐bearing mice.^[^ ^365^ ^]^

miRNA controls almost all basic biological processes of organisms, including autophagy.^[^ ^366^ ^]^ miR‐388‐5p inhibited autophagy by downregulating the expression of target genes PIK3C3 and Vps34, thereby enhancing the migration of colorectal cancer cells.^[^ ^367^ ^]^ miR‐17 increases the sensitivity of tumor cells to chemotherapy drugs and low‐dose ionizing radiation treatment via interfering with the ATG7 gene.^[^ ^368^ ^]^ The downregulation of microRNA miR‐23b increased the expression of target gene ATG12 and autophagy activity in pancreatic cancer cells, thereby promoting radiation resistance.^[^ ^369^ ^]^ lncRNA is a class of RNAs with a length of more than 200 nucleotides with low protein‐coding ability.^[^ ^370^ ^]^ As an important signaling molecule in vivo, lncRNA is involved in transcriptional silencing, transcriptional activation, chromatin modification, and nuclear transport.^[^ ^371^ ^]^ It has been suggested that lncRNA could regulate multiple autophagy genes at the same time, thereby regulating different stages of the autophagy process.^[^ ^372^ ^]^ LncRNA could bind certain miRNAs and act as competing endogenous RNA to regulate the expression of relevant miRNA target genes and coregulate autophagy. Yang et al. demonstrated that the lncRNA PVT1 interacts with microRNA‐365 to regulate ATG3, affecting autophagy.^[^ ^373^ ^]^ Similarly, Liu et al. showed that lncRNA SNHG15 is highly expressed in OS cells and negatively regulates miR‐141 by acting as a molecular sponge for miR‐141, thus affecting autophagy and proliferation in OS cells.^[^ ^374^ ^]^ Luo et al. reported that EIF3J‐DT and ATG14 are highly expressed, chemotherapy‐resistant gastric cancer patients. lncRNA EIF3J‐DT activated autophagy and induced drug resistance by targeting ATG14, making it a potential therapeutic target to enhance chemosensitivity and improve prognosis. EIF3J‐DT binds and stabilizes ATG14 mRNA, preventing its degradation by competing with (miRNA) miR188‐3p.^[^ ^375^ ^]^ Since the lncRNA‐miRNA axis is widespread in tumors, it is suggested that autophagy is likely to be controlled by this axis and thus act on tumors. CircRNAs are a special class of noncoding RNA molecules formed by reverse splicing.^[^ ^376^ ^]^ circRNA can affect miRNA expression together with lncRNA.^[^ ^377^ ^]^ Since miRNA has binding sites for both lncRNA and circRNA, their expression can be regulated by sponge effects in cancer.

Although studies have shown that delivering ncRNAs may silence autophagy‐related genes to overcome drug resistance in cancer,^[^ ^378^, ^379^, ^380^ ^]^ research is still in its early stages, the same could be said about other nucleic acid drugs for cancer therapy in general. However, progress has been made to overcome existing challenges related to stability, delivery, efficacy, safety, and manufacturing, bringing the clinical application closer.^[^ ^362^ ^]^

### 4.8 Natural Products

Natural compounds form the basis of many anticancer drugs, with over 50% of cancer drugs derived from natural substances.^[^ ^381^ ^]^ A growing number of studies have shown that natural compounds can regulate autophagy in tumor cells, including matrine,^[^ ^382^ ^]^ triterpenoid saponin,^[^ ^383^ ^]^ oblongifolin C,^[^ ^384^ ^]^ rhizochalinin,^[^ ^385^ ^]^ epigallocatechin gallate,^[^ ^386^ ^]^ and elaiophylin.^[^ ^387^ ^]^ Natural anticancer compounds could be used to either induce autophagy in tumor cells to cause cell death and inhibit metastasis or inhibit autophagy to enhance tumor sensitivity to drugs. Some compounds even exert opposing effects, depending on the type of cancer.^[^ ^388^ ^]^ Most natural products could be integrated into nanodelivery systems. For instance, the nanodelivery of solid lipid curcumin particles overcomes the shortcomings of poor water solubility and chemical instability. It can activate the expression of autophagy protein beclin‐1, inhibit the PI3K‐Akt/mTOR pathway, and block glioblastoma progression.^[^ ^389^ ^]^ As discussed above, the autophagy‐inducing properties of curcumin have been exploited to trigger ACD in tumors in combination with PTT or BMS1166.^[^ ^194^, ^328^ ^]^ Although many current natural anticancer compounds can modulate autophagy, the interactions between autophagy and other cellular processes and cell death pathways are sometimes unclear. For instance, curcumin, a known autophagy regulator, has antioxidant, anti‐inflammatory, and antitumor properties.^[^ ^390^ ^]^ Therefore, elucidating the mechanism of autophagy modulation by natural compounds will help develop more specific autophagy modulators.

## 5 Clinical Trials for Autophagy‐Modulating Drugs

This section reviews clinical trials using drugs explicitly targeting autophagy as inhibitors or inducers. Given the lack of nanomedicines targeting autophagy in clinical trials, this section serves as a starting point to understand the challenges of existing therapies and identify opportunities that could be overcome by nanomedicine.

### 5.1 Autophagy Inhibitors

Most clinical trials targeting autophagy in cancer have focused on autophagy inhibition, using CQ and HCQ as monotherapy (1/3) or combined with chemotherapy, radiotherapy, or immunotherapy (2/3).^[^ ^1^ ^]^ Recently, selective inhibitors such as DCC‐3116 (ULK inhibitor) and GNS561 (PPT1 inhibitor) are being tested. Over 90% of trials are in phase I/II stage for solid tumors.^[^ ^1^, ^391^, ^392^, ^393^, ^394^ ^]^ No efficacy was shown in monotherapy, and no autophagy inhibitor has been approved for clinical use.^[^ ^395^, ^396^ ^]^ Combination strategies with chemotherapy/radiotherapy have shown some success but remain inconsistent. Reported combinations include taxanes (DTX, PTX, Abraxane), GEM, TMZ, cyclophosphamide, carmustine, ixabepilone, proteasome inhibitor (bortezomib), multikinase inhibitor (regorafenib), HDAC inhibitors (vorinostat and entinostat, EGFR TKI (erlotinib), mTOR inhibitor (rapamycin, everolimus, and sirolimus), immunotherapy drug (aldesleukin, antibodies (bevacizumab and avelumab), and metformin.^[^ ^391^ ^]^

Notably, CQ, added to glioblastoma standard treatment, extended the median survival (33 vs. 11 months) and reduced mortality in the phase III trial (NCT00224978).^[^ ^397^, ^398^ ^]^ It was well tolerated with RT/TMZ or whole‐brain radiation therapy in high‐grade glioma patients (NCT02378532, NCT01727531).^[^ ^399^, ^400^ ^]^ In contrast, HCQ with radiotherapy/temozolomide showed no significant survival advantage (NCT00486603).^[^ ^401^ ^]^ CQ with taxanes improved the objective remission rate (ORR) (45% vs. 30%) in anthracycline‐refractory breast cancer (NCT01446016),^[^ ^402^ ^]^ and reduced proliferation and immune cell migration in breast ductal carcinoma in situ (NCT01023477),^[^ ^403^ ^]^ but preoperative CQ had no effect in breast cancer (NCT02333890).^[^ ^395^ ^]^ HCQ with PTX and carboplatin had a modest effect on metastatic NSCLC regardless of the Kras‐mutation status (NCT00728845, NCT01649947).^[^ ^404^ ^]^ High‐dose HCQ with TMZ prolonged stable disease and partial responses in melanoma patients.^[^ ^405^ ^]^ Ongoing trials are evaluating HCQ with nivolumab or nivolumab/ipilimumab in stage III–IV melanoma (NCT04464759). The efficacy of autophagy inhibition in pancreatic cancer remains limited despite showing good tolerability and improvement of tumor biology. CQ with GEM showed potential (NCT01777477), but preoperative HCQ with Abraxane and GEM did not improve overall survival and relapse‐free survival rates in resectable pancreatic adenocarcinoma despite decreasing CA19–9 levels and showing evidence of autophagy inhibition (NCT01978184).^[^ ^406^, ^407^ ^]^ Similarly, no 12‐month survival benefit was observed for GEM and nab‐PTX (NCT01506973). In refractory multiple myeloma, CQ with bortezomib/cyclophosphamide showed a partial response^[^ ^408^ ^]^ while HCQ improved the response to bortezomib/cyclophosphamide in pretreated patients (NCT00568880).^[^ ^409^ ^]^ In renal cell carcinoma, HCQ with everolimus (mTOR kinase inhibitor) achieved stable disease in two‐thirds of patients with a 45% 6‐month progression‐free survival (PFS) (NCT01510119);^[^ ^410^ ^]^ and encouraging results were obtained with IL‐2 (aldesleukin) (17 months PFS) (NCT01550367).^[^ ^411^ ^]^ In colorectal cancer, HCQ showed no benefit with entinostat/regorafenib (NCT03215264),^[^ ^412^ ^]^ and HCQ/Vorinostat performed worse than regorafenib alone (NCT02316340).^[^ ^413^ ^]^ HCQ with mFOLFOX6/bevacizumab had a 68% response rate and 74% one‐year overall survival; however, withdrawal rates were high (NCT01206530).^[^ ^414^ ^]^ CQ with metformin showed no response in IDH1‐mutated solid tumors (NCT02496741).^[^ ^415^ ^]^

Finally, DCC‐3116, a potent ULK inhibitor, showed preclinical activity with MAPK inhibitor trametinib. A phase 1 trial is ongoing for DCC‐3116 in monotherapy and in combination with other RAS/MAPK inhibitors (trametinib, binimetinib, or sotorasib) in patients with advanced or metastatic solid tumors harboring RAS/MAPK mutations (NCT04892017). This may position ULK as the first autophagy‐specific target in the clinic.^[^ ^294^ ^]^ PPT1 inhibitor GNS561 has shown safety (NCT03316222),^[^ ^416^ ^]^ and phase 1/2 trial with trametinib in KRAS‐mutant cholangiocarcinoma is ongoing (NCT05874414).

### 5.2 Autophagy Inducers

A few autophagy inducers are being explored as potential cancer therapies due to their ability to promote cell death in tumor cells. ABTL0812 induces autophagy via PPARα/γ and Akt/mTOR, although it has been suggested that the cell death may be primarily autophagy independent.^[^ ^294^ ^]^ Pevonedistat, a NEDD8‐activating enzyme inhibitor, triggers cell‐cycle arrest, apoptosis, senescence, and autophagy in many cancer cells.^[^ ^417^ ^]^ Clinical trials in solid tumors, melanoma, acute myeloid leukemia, and myelodysplastic syndromes have shown that Pevonedistat is generally well tolerated with promising overall survival, PFS, and ORR results. ABTL0812 has completed phase 1/2 trial in combination with PTX and carboplatin in patients with advanced/recurrent endometrial cancer, showing a good safety profile and encouraging activity (NCT03366480).^[^ ^418^ ^]^ Other autophagy inducers, such as rapamycin and everolimus, have been shown to enhance the antitumor efficacy of chemotherapy.^[^ ^410^, ^419^ ^]^ Nab‐sirolimus (albumin‐bound rapamycin) was approved by the FDA in late 2021 (Fyarro, Aadi Bioscience, Inc.) for locally advanced unresectable or metastatic malignant perivascular epithelioid cell tumor (PEComa).^[^ ^410^, ^419^ ^]^ A phase II trial reported an ORR of 38.7%, with a median duration of response of 39.7 months and a median overall survival of 53.1 months (NCT02494570).^[^ ^420^, ^421^ ^]^ Previously, Nab‐sirolimus was well tolerated at 100 mg m^−2^ weekly for 4 weeks (NCT03817515, NCT03190174, NCT00635284, NCT02646319) and exhibited potent inhibition of mTOR targets, S6K and 4EBP1 in patients with unresectable and metastatic solid malignancies.^[^ ^422^ ^]^ Other clinical trials are under way for metastatic colorectal cancer, advanced sarcomas, and pediatric patients with refractory primary central nervous system cancers.

## 6 Challenges of Current Autophagy Modulators

CQ/HCQ have been used for decades and are considered safe with a low risk/benefit balance as antimalarial drugs, especially with short‐term usage.^[^ ^423^, ^424^ ^]^ HCQ is more soluble and considered safer than CQ. Various phase I/II studies showed that CQ/HCQ is well‐tolerated by cancer patients in mono and combination therapies. Maximal tolerated doses up to 600–1200 mg daily were reported for HCQ when combined with radiation therapy and TMZ (NCT00486603),^[^ ^401^, ^405^ ^]^ regorafenib and entinostat (NCT03215264),^[^ ^412^ ^]^ GEM (NCT01128296),^[^ ^425^ ^]^ everolimus (NCT01510119),^[^ ^426^ ^]^ erlotinib (NCT 00977470), and cyclophosphamide/dexamethasone (NCT01396200). The poor antitumor efficacy of CQ/HCQ may stem from insufficient activity in monotherapy and inadequate autophagy suppression within tumors.^[^ ^391^ ^]^ CQ/HCQ have excellent oral absorption and bioavailability (0.7–0.9) and a very long elimination half‐life (days to weeks).^[^ ^427^, ^428^ ^]^ However, the large volumes of distribution may limit their significant accumulation in tumors. Moreover, the acidic tumor environment helps protonate CQ/HCQ (weak bases), reducing cell permeability and efficacy.^[^ ^429^ ^]^ Furthermore, the efficacy of CQ/HCQ was mostly absent or unsatisfactory in several clinical trials despite using high doses reaching dose‐limiting toxicities.^[^ ^395^, ^401^, ^409^ ^]^ Therefore, finding more potent and safer CQ/HCQ derivatives/alternatives and improving accumulation within tumors through passive or active targeting are worth exploring.^[^ ^110^, ^430^ ^]^

Most current small‐molecule autophagy inhibitors are not specific to autophagy and can affect multiple cellular pathways, which may lead to off‐target effects. For instance, the lysosomal inhibitors CQ and HCQ have multiple cellular targets, which may limit their clinical application.^[^ ^431^, ^432^ ^]^ Some PI3K inhibitors, such as 3‐MA, LY294002, and wortmannin, can affect class I and III PI3K activities, complicating their role in autophagy regulation.^[^ ^391^ ^]^ 3‐MA has a dual role in autophagy as it inhibits autophagy by inhibiting Vps34 but could also suppress class I PI3K activity, leading to autophagy induction through the PI3K/Akt/mTOR pathway.^[^ ^391^, ^433^ ^]^ ULK1/2 inhibitors, such as MRT68921, also inhibit AMPK and TANK‐binding kinase 1 activities.^[^ ^391^, ^434^ ^]^ For a more comprehensive discussion, readers are referred to detailed reviews.^[^ ^391^, ^435^ ^]^

Similarly, the lack of potency of some autophagy modulators, whether inherent or due to delivery issues (e.g., poor solubility, stability) or PK properties, may affect their ability to reach tumor sites at effective concentrations. Rapamycin, for instance, despite being a potent mTOR inhibitor, suffers from poor solubility and suboptimal PK. These limitations led to the development of albumin‐bound formulations (Nab‐sirolimus) and second‐generation mTOR inhibitors such as Everolimus and Temsirolimus.^[^ ^435^ ^]^

Developing potent and selective autophagy modulators is challenging as autophagy‐related proteins are not easily druggable. Yet, several inhibitors have been developed for ULK1/2, VPS34, V‐ATPase, and PPT1. ULK1/2 is a key player in the early stages of autophagy and has potentially druggable serine/threonine kinase. Its inhibitors include SBI‐0206965 and its analog, SBP‐7455, ULK‐100, ULK‐101, MRT67307, MRT68921, and DCC‐3116.^[^ ^294^ ^]^ The latter is in phase 1/2 trials for RAS/RAF‐mutated solid tumors (NCT04892017). VPS34 inhibitors such as SAR405 and SB02024 exist.^[^ ^436^ ^]^ 249C inhibits autophagy and micropinocytosis by blocking V‐ATPase activity, thus preventing lysosomal acidification.^[^ ^437^ ^]^ PPT1 regulates lysosomal acidification by controlling the subcellular localization of V‐ATPase.^[^ ^438^ ^]^ It has been reported as the target for CQ/HCQ derivatives Lys01, dimeric quinacrines, long‐linkered dimeric CQs, and GNS561.^[^ ^416^, ^439^ ^]^ This later is in phase 1/2 clinical trials (NCT05874414). BAQ derivatives, combining lysosomotropic detergent (MSDH) and autophagy inhibitor Lys05, are potent autophagy inhibitors that can self‐assemble into NPs, but their molecular targets are still unknown.^[^ ^110^, ^111^ ^]^

AUTOphagy‐TArgeting Chimera is a new technology that can selectively degrade proteins through the autophagy‐lysosome system and is leveraged for cancer therapy.^[^ ^440^ ^]^ It employs bifunctional molecules with target‐binding ligands linked to autophagy‐targeting ligands. The target‐binding ligands bind to target proteins (i.e., oncoproteins) and drag them to the ZZ domain autophagy receptor p62/Sequestosome‐1/SQSTM1, which is then activated, leading to target protein sequestration and degradation.

Many FDA‐approved drugs have been considered for drug repurposing for their autophagy‐modulating and antitumor properties.^[^ ^441^ ^]^ For instance, chlorpromazine was found to trigger cytotoxic autophagy in glioblastoma cells through ER stress and is currently being evaluated in clinical trials for GBM patients with hypo‐ or unmethylated MGMT gene, which have intrinsic resistance to temozolomide.^[^ ^442^ ^]^ Repurposed drugs have a known safety/toxicity profile, may benefit from reduced cost and development time, and offer improved access to patients. However, challenges including potential adverse side effects due to multiple mechanisms, especially at potentially higher doses, limited efficacy as single agents, lack of patent incentives, and regulatory hurdles, may arise.^[^ ^441^, ^443^ ^]^

As autophagy modulators are often evaluated with other cancer treatments (e.g., chemotherapy, immunotherapy, or radiation), the timing, dose, and schedule of combination therapy add another layer of difficulty in designing the clinical trials. For example, CQ/HCQ have been given in preoperative and postoperative settings.

Another challenge facing autophagy inhibitors is finding a reliable biomarker for accurately measuring autophagic activity in tumors. While LC3‐II and SQSTM1/p62 levels in peripheral blood have been used as proxies for autophagy in tumor cells, their utility is not without limitations.^[^ ^391^ ^]^ For example, in patients with early‐stage solid tumors treated with HCQ, the secretion of Par‐4 correlated with apoptosis in the cancer but did not correlate with the autophagy inhibition marker p62 (NCT03015324), highlighting the need for more precise and tumor‐specific biomarkers to evaluate autophagic activity and its therapeutic implications effectively.^[^ ^391^ ^]^

Several other limitations related to autophagy and cancer biology, in general, include 1) the complexity of signaling pathways involved in autophagy and their interconnectedness with other pathways regulating apoptosis and cell survival, 2) different outcomes of autophagy modulation at various stages of cancer development, 3) the variability among different cancer types, and 4) variability within different cancer regions, subtypes, microenvironments, as well as differences between metastatic and primary sites. In addition, autophagy levels and activity can vary significantly between different tumors and within the same tumor, further complicating therapeutic approaches.

## 7 Conclusion and Perspectives

The dual role of autophagy as both tumor suppressor and promoter poses significant challenges in developing effective cancer treatments, as modulating autophagy may have opposing effects on tumor growth and treatment outcomes. Current strategies in cancer therapy often involve autophagy inhibition to sensitize cancer cells and overcome treatment resistance, typically in combination with chemotherapy, radiotherapy, and immunotherapy.

Classic autophagy inhibitors such as CQ and HCQ have shown good safety profiles in clinical trials but have limited efficacy as monotherapies. Their effectiveness in combination therapies remains inconsistent, and no autophagy inhibitor has yet received approval for cancer therapy.^[^ ^444^ ^]^ Furthermore, no nanoformulation encapsulating HCQ/CQ has been tested in clinical settings either alone or in combination with other drugs. In contrast, autophagy inducers, such as the mTOR inhibitor Nab‐sirolimus, have been clinically approved. However, their mechanism of action is not specific to autophagy, and there are concerns that autophagy induction may promote accelerated tumor growth in advanced cancers.^[^ ^294^ ^]^

Limitations of the current small‐molecule autophagy modulators, such as solubility issues, suboptimal PK, off‐target toxicities, and poor tumor accumulation, could potentially be addressed using nanomedicine strategies (see Table 1). Recent candidates such as DCC‐3116 and GNS561 have shown good oral bioavailability;^[^ ^445^, ^446^ ^]^ however, issues of poor aqueous solubility and low absorption may hinder the clinical translation of novel selective autophagy modulators.^[^ ^447^, ^448^, ^449^ ^]^ This could be addressed using nanoformulations for parenteral injection. Additionally, other nanocarrier‐based strategies, such as self‐nanoemulsifying drug delivery systems and LNPs, can improve the oral bioavailability of poorly soluble and permeable drugs; however, their benefits are largely limited to enhancing bioavailability without providing advantages like improved tumor accumulation and targeting, which are often associated with nanomedicine.

Challenges related to the lack of potency and selectivity can be, to some extent, addressed using nanoplatforms through tissue targeting and stimuli–responsive release. For example, the on‐demand autophagy‐triggered release strategy in which the drug is only offloaded from NPs in the case of autophagy dysregulation represents a promising approach for achieving precise therapeutic control.^[^ ^108^ ^]^ NRs designed to target and degrade oncogenic proteins via autophagy represent another promising strategy.^[^ ^107^ ^]^ These strategies could also enable theranostic applications, allowing autophagy‐specific imaging and monitoring.

Many NMs tend to accumulate in lysosomes, making them effective tools for targeting autophagy via the lysosomal pathway. Cancer cells tend to have more fragile lysosomes than healthy normal cells and, therefore, are more susceptible to LMP. Several NPs have been reported to accumulate in lysosomes and induce LMP.^[^ ^109^, ^110^, ^111^, ^450^ ^]^ These NPs often cause lysosomal swelling through the proton‐sponging effect, where weak bases protonate in the acidic lysosomal environment, elevating the lysosomal pH and impairing autophagic function. Alternative mechanisms, such as NP aggregation or nanofibril transformation within lysosomes, have also been reported.^[^ ^109^, ^111^ ^]^ These approaches could be tweaked by incorporating other lysosomal‐targeting and pH‐responsive units (ligands, weak bases such as morpholine and aminoquinolines, lysosomotropic detergents, and peptides). Ionizable lipids, widely used in LNPs for mRNA delivery, present another opportunity. These lipids are designed to protonate at low pH and promote endosomal escape by interacting with the negatively charged phospholipids of the endosomal membrane.^[^ ^451^, ^452^ ^]^ Given their proven efficacy in intracellular mRNA delivery, designing new ionizable lipids with lower pKa could delay their protonation and direct them toward lysosome disruption rather than endosomal escape.

Other forms of specialized autophagy mechanisms, such as the selective degradation of mitochondria (mitophagy), ER (ER‐phagy), and lysosomes (lysophagy), are being studied and could provide new opportunities for developing new autophagy modulators.^[^ ^449^ ^]^ Several small molecules have been identified to target these pathways, including WJ460, FL3, and Fluorizoline for mitophagy; Brigatinib, C150, ABTL0812, and Loperamide for ER‐phagy; Resveratrol and Tigecycline for xenophagy; Tripterine and PFK158 for lipophagy; and Pimozide and GNS561 for lysophagy.

Combining autophagy modulators with other therapeutic agents, such as chemotherapy and immunotherapy, phototherapy, gene therapy, and imaging agents, can enhance therapeutic efficacy by targeting multiple pathways. While combinations with chemotherapy and radiotherapy dominate clinical trials, increasing attention is given to immunotherapy. Notably, ongoing trials combining HCQ and immunotherapeutic agents (administered separately, not as NPs), including Aldesleukin (IL‐2), nivolumab (anti‐PD‐1) alone, or in conjunction with Ipilimumab (anti‐CTLA‐4) and Avelumab (anti‐PD‐L1), are currently under way. The ability of autophagy modulators to enhance immune responses and overcome drug resistance positions them as valuable additions to immune checkpoint blockade and other immunotherapies.

Despite numerous preclinical studies on NPs for autophagy modulation, their clinical translation remains limited. Most clinical trials rely on conventional oral formulations, when possible, due to lower cost, simplicity, and faster development timelines, while maintaining the flexibility to integrate with established therapeutic regimens. In contrast, nanomedicine is reserved for more challenging delivery scenarios. Combining autophagy modulators with other drugs within the same physical NP remains largely untested in clinical trials.

In terms of NP design, lipid‐based NPs (i.e., liposomes, LNPs, nanoemulsions), polymeric, albumin‐conjugated, and iron oxide are well accepted and exhibit favorable safety profiles. Moreover, they can accommodate drugs with different properties (hydrophobic/hydrophilic and biologics), have good manufacturability, and are approved by the FDA, making them adequate candidates for fast clinical applications. More advanced nanocarriers usually offer enhanced functionalities such as active targeting and controlled and stimuli–responsive release; however, their complex designs, often high production costs, and the use of novel materials pose additional challenges related to stability, safety (e.g., metallic, carbon‐based, novel polymers), manufacturability, and regulatory approval.

Nanomaterials with intrinsic autophagy‐modulating properties have generally promiscuous mechanisms of action and induce cell death via multiple pathways. While this might be beneficial, it could lead to off‐target effects and unpredictable toxicity profiles. Furthermore, many of these materials lack clinical validation. Other systems, such as exosomes, are worth exploring for their potential ability to modulate autophagy, serve as carriers for autophagy‐targeting drugs, and selectively target some tissues.^[^ ^453^ ^]^

Nanomedicine offers new opportunities for delivering natural compounds and repurposed drugs for autophagy modulation by improving PK and tumor accumulation. These advancements can enhance the efficacy of these drugs with the incentive of generating new intellectual property.

While advances in autophagy‐based nanotherapies are promising, several challenges remain. The potential toxicity of nanocarriers remains a concern, necessitating comprehensive studies to evaluate their safety profiles in human applications. Additionally, overcoming biological barriers (e.g., BBB, TME) and achieving extrahepatic delivery remain broader challenges for nanomedicine. Finally, as advanced therapeutic modalities such as CRISPR‐Cas9 and nucleic acids are maturing and nanoformulations such as liposomes, LNPs, and polymeric NPs become more feasible, nanomedicine is increasingly seen as the key to unlocking the full potential of these therapies. This trend is expected to expand to include combination strategies and be incorporated into the early stages of drug development.

In summary, autophagy presents both challenges and opportunities in cancer therapy. Nanocarriers hold significant potential for improving the delivery, efficacy, and safety of autophagy modulators. However, addressing the existing challenges, such as the need for improved autophagy inhibitors, better targeting strategies, and safer nanocarriers, is essential for realizing the full potential of this approach in clinical practice. Continued research in these areas could pave the way for transformative advancements, improving cancer patients’ survival and quality of life.

## Conflict of Interest

The authors declare no conflict of interest.

## Author Contributions

**Sohaib Mahri**: conceptualization (lead); writing—original draft (lead); writing—review & editing (lead). **Rodolfo Villa**: writing—original draft (supporting); writing—review & editing (supporting). **Ya‐Ping Shiau**: writing—original draft (supporting). **Menghuan Tang**: writing—original draft (supporting). **Kelsey Jane Racacho**: visualization (supporting); writing—original draft (supporting). **Qiufang Zong**: writing—original draft (supporting). **Saiful Islam Chowdhury**: writing—original draft (supporting). **Tan Hua**: writing—original draft (supporting). **Felipe Godinez**: funding acquisition (lead). **Andrew Birkeland**: funding acquisition (lead). **Tzu‐Yin Lin**: funding acquisition (supporting); investigation (lead). **Yuanpei Li**: funding acquisition (lead); investigation (lead); writing—review & editing (lead).
